# Status, barriers and facilitators of comprehensive abortion care services in rural India: A multicenter mixed-methods study protocol

**DOI:** 10.1371/journal.pone.0358274

**Published:** 2026-09-16

**Authors:** Subhanwita Manna, Bijaya Kumar Mishra, Krishna Kant Yadav, Pratibha Dhiman, Smiteerekha Sahoo, Chiranjeet Mishra, Tanveer Rehman, Ranjan Kumar Prusty, Ragini Kulkarni, Mahadev Bhise, Ashok Kumar Pandey, Ravikumar B. S., Ipsita Pal Bhowmick, Subrata Kumar Palo, Narayana Swamy D. M., Neha Srivastava, Umaer Alam, Reeta Singh, Manish Barvaliya, Vani Kandpal, Barsha Gadapani Pathak, Reema Mukherjee, Sanghamitra Pati

**Affiliations:** 1 Division of Reproductive, Child Health & Nutrition, ICMR, New Delhi, India; 2 ICMR-National Institute of Health Research, Bhubaneswar, Odisha, India; 3 Society for Applied Studies, New Delhi, Delhi, India; 4 ICMR-National Institute For Research in Reproductive and Child Health, Mumbai, India; 5 ICMR-National Institute of Health Research, Gorakhpur, Uttar Pradesh, India; 6 ICMR – National Institute of Traditional Medicine, Belagavi, Karnataka, India; 7 ICMR-Regional Medical Research Centre, Dibrugarh, Assam, India; University College of Medical Sciences, INDIA

## Abstract

**Background:**

Unsafe abortion continues to be a significant cause of avoidable maternal morbidity and mortality especially in low- and middle-income countries. Despite India’s permissive Medical Termination of Pregnancy (MTP) Act, operational gaps continue to prevent women from accessing safe abortion services. There is still a dearth of multi-centric mixed-methods research from rural areas on women’s experiences, provider perspectives, and facility readiness. This study aims to assess the status of comprehensive abortion care (CAC) in rural India by evaluating public-sector facility preparedness, provider experiences, care-seeking pathways, and identifying key barriers and facilitators as per CAC guidelines (2023). The findings will guide the development of a preliminary implementation approach (Model 0+) as well as future phases focused on testing, adaptation, and scale up to strengthen CAC availability, quality, and uptake.

**Methods:**

This study employs a convergent mixed-methods design across six geographically varied rural blocks in India (North, South, East, West, Central, and Northeast). There will be two participant groups: married women aged 18–49 years who are current, previous, or potential abortion service users, and public health system stakeholders involved in Comprehensive Abortion Care (CAC). A multistage cluster survey will enroll approximately 854 married women per site across 20 clusters. The qualitative component will comprise interviews with women who have had abortions in the last two years and healthcare practitioners, for a total of around 240 interviews. Additionally, 18 focus group discussions with Accredited Social Health Activists and Anganwadi Workers (three per site), will capture frontline worker’s perspectives. Quantitative data will be collected using KoboCollect and analysed using STATA (or an equivalent statistical software). Qualitative data will undergo combined phenomenological and thematic analysis using NVivo or equivalent software.

**Discussion:**

This study constitutes the first phase of a multi-stage implementation research programme. The study builds upon national CAC guidelines as a foundation of implementation of “Model 0” for abortion care and assesses their functioning in routine public-sector settings. Insights from women, frontline workers, and providers will inform development of a refined implementation strategy (“Model 0+”) grounded in real-world contexts. This evidence-driven approach is expected to yield a practical, acceptable, and scalable model for subsequent piloting, adaptation, and wider health system uptake.

## Introduction

Availability and access to safe abortion services is a critical component of sexual and reproductive health and a key determinant of maternal health outcomes particularly in rural areas. Globally, unsafe abortion continues to contribute substantially to preventable maternal morbidity and mortality, with the burden disproportionately concentrated in low- and middle-income countries [1–3]. Barriers related to health system capacity, facilitators availability, social stigma, and legal and policy misinterpretation often limit women’s ability to obtain timely, safe, and respectful abortion care, particularly in rural and underserved settings [4,5].

India has a comparatively liberal legal framework for abortion under the Medical Termination of Pregnancy (MTP) Act, originally enacted in 1971 and subsequently amended to expand gestational limits and eligible provider categories [6,7]. To operationalize the law, the Ministry of Health and Family Welfare (MoHFW) has issued comprehensive abortion care (CAC) guidelines, most recently updated in 2018 and 2023, which outline standards for service provision across different levels of public health system, including Primary Health centers, community health centers, and higher referral facilities [8,9]. These guidelines are developed by a set of evidence-based practices, including decentralization of abortion services at lower-level facilities, task-sharing with trained providers, provision of medical abortion at primary care level, standardized provider training, and delivery of non-judgemental, women-centered counselling services. Evidence from India and other low-and-middle-income settings indicates that these evidence-based practices are effective in improving access to safe abortion services when implemented as intended [10,11]. Studies from western India found that, CAC model increased facility availability from 0% to 20% in the intervention area along with improved providers’ awareness of women’s abortion-related rights, a rise in the display of IEC materials from 2% to 93% of PHCs, and improvements in essential CAC equipment of up to 12%. The study further noted that the expansion of facilities providing CAC services was associated with an increase in abortions conducted at health facilities [12].

However, these improvements underscore a critical insight: the existence of sound policy and technical guidance alone does not guarantee consistent service delivery. The persistent gaps in facility readiness, provider behaviour, and women’s access pathways reflect a systemic implementation gap rather than a policy deficit. The central challenge therefore lies not in the absence of policy, but in the implementation of these guidelines in routine practice. Gaps in facility readiness, provider practices and women’s care seeking pathways are best understood as interconnected dimensions of a broader implementation gap between CAC policy intent and on the ground service delivery.

Despite enabling the policy environment, evidence consistently indicates that access to quality CAC services within the public sector remains uneven [13]. Rural areas, in particular, continue to face challenges related to facility readiness, shortages of trained providers, inconsistent availability of drugs and equipment, and limited awareness among women regarding the legality and availability of abortion services [14,15]. As a result, many women continue to seek abortion from informal or unregulated providers, increasing the risk of complications and adverse outcomes [16]. Existing studies on abortion care in India have largely focused on utilization patterns, incidence and methods of abortion, or legal awareness among women [15,16]. India has made substantial policy commitments to expand access to safe abortion care, including the roll out of comprehensive national guidelines and provider training initiatives. However, the effectiveness of these efforts ultimately depends on whether women actually utilize CAC services from trained providers within the public health system. If services are available but not accessed, policy intent does not translate into meaningful health impact. Despite this, there is comparatively limited systematic evidence analyzing the implementation of CAC services within public health facilities, especially across diverse rural contexts. In particular, there is a lack of integrated assessments that simultaneously examine service availability and readiness, provider perspectives on service delivery, and women’s experiences of seeking and accessing care. Furthermore, few studies explicitly analyze how national CAC guidelines translate into routine practice at the facility level, or where gaps arise between policy intent and on-the-ground implementation [17]. Implementation research is therefore essential to address these gaps.

For improving the quality of health services to rural population in the states, Government of India established model rural health and research units (MRHRUs) with a tripartite partnership between Indian Council of Medical Research (ICMR) institutes, Medical Colleges and Directorate of Health Services at State level. It strengthens and bridges the gap between modern medical technology and healthcare delivery in rural areas [18]. Our study conducted in the selected rural blocks across varied geographic and health system contexts, will generate context-specific evidence, particularly within resource-constrained areas. Evidence generated through this mixed-method study can inform adaptive strategies that are both locally relevant and scalable within the National Health system.

There is currently limited multi-centric, mixed-methods evidence from rural India that simultaneously examines facility readiness, provider perspectives and women’s care-seeking experiences to understand how national CAC guidelines are implemented in routine public sector practice. To bridge this gap, the present study aims to assess the status of comprehensive abortion care services through systematic assessment of facility readiness, provider experiences, and women’s care-seeking pathways and identify the barriers and facilitators to their provision and uptake, in order to develop an implementation strategy (Model 0+) for improving CAC in rural India. Findings from this phase will inform subsequent phases focused on piloting, adaptation, and scale-up of interventions to improve the availability, quality, and uptake of CAC services within the public health system.

### Objectives

The specific objectives are

(i) To document the availability and functionality of CAC services across different levels of public health facilities in the selected MRHRU areas.
(ii) To explore service provider-level barriers and facilitators related to delivery of CAC services.
(iii) To evaluate women’s awareness of abortion services, attitude towards abortion, experiences in seeking care, socio-cultural barriers and facilitators influencing the uptake of CAC services.
(iv) To utilize the study findings to develop a contextually grounded implementation model (Model 0+) for improving CAC services, to be piloted in the next phase of this study.

## Methodology

### Study design

This study will use a convergent (also known as concurrent or parallel) mixed-methods design [19]. The design involves the collection of qualitative and quantitative data separately and then merges the result at the time of interpretation where they are analyzed side-by-side. The intent of using this designs is to compare both quantitative and qualitative data gathered at the same time to identify correlations between relevant variables [20]. In the quantitative part, data will be collected at two levels. First, individual-level data on women’s knowledge, awareness, experiences, and attitudes related to abortion services will be collected using a structured questionnaire through a household survey. Second, facility-level data on the preparedness of public health facilities to provide CAC services will be collected using a standardized facility observation checklist. In the qualitative part, semi-structured interviews will be conducted with selected women and key health system stakeholders to explore in depth their perspectives on abortion service delivery, acceptability, facilitators, and barriers. Findings from both the qualitative and quantitative parts will be integrated to develop the Model 0 + .

### Study setting

The study will be conducted in rural communities and associated health facilities in the catchment area (i.e., blocks) of MRHRUs of Odisha, Jharkhand, Maharashtra, Karnataka, Uttar Pradesh and Tripura. The study will be multi-centric, conducted in six model rural health research units (MRHRU) areas selected to cover diverse regions of India, covering North, South, East, West, Central, and Northeast zones. These MRHRUs were purposively selected based on geographic representation, research readiness, and operational feasibility. The selected MRHRUs include Tigiria (Odisha), Namkum (Jharkhand), Dahanu (Maharashtra), Raichur, (Karnataka), Pali (Uttar Pradesh), and Khumulwng (Tripura). The study will be implemented in MRHRU’s designated study block. These blocks represent predominantly rural and tribal settings with populations ranging from approximately 70,000–402,095 individuals. The details of the study blocks are represented in Table 1.

**Table 1:** Details of study sites.

Name of MRHRU and Study Block (District, State)	Area Type (Urban/Rural/Tribal/Mixed)	Approx. Population of Block	No. of Villages in Study Block	Public Health Facilities Included*	Referral Facility Available (FRU/CEmONC)	Private Abortion Providers Present (Yes/No; approx.)	Facilities Providing Abortion Services†
Tigiria (Cuttack, Orissa)	Mixed, predominantly rural	76379	48	1 DH, 1 SDH, 2 CHC, 4 PHC, 7 HWC	Yes	Yes	Yes
Namkum (Ranchi, Jharkhand)	Rural	1,57,455	196	1 CHC, 1 PHC, 25 AAMs.	Yes; CEmONC	Yes; approximately 3	AAMs- Medical abortion CHC- Medical and/or surgical abortion Private facility-3 (provides both Medical and/or surgical abortion)
Dahanu (Palghar, Maharashtra)	Rural and Predominantly tribal	402,095	173	2SDHs 1 Rural Hospital/CHC 9 PHCs 65 SCs/HWCs	Yes	Yes	
Khumulwng(West Tripura District Tripura)	Tribal /Rural (Mixed)	34000	15	1 CHC, 1 PHC 15 SCs		Yes	No
Pali, Uttar Pradesh	Rural	120013	138	1 CHC, 3 PHC, 29 Sub Centre/HWC	Yes	Yes	Yes
MRHRU, Sirwar, Raichur, Karnataka	Rural	1,69,962	92	1 CHC 3 PHC 20 SC	Yes	Yes	No

CHC- Community Health Center, PHC- Primary Health Center, SC- Sub Center, HWC- Health and Wellness Centres, RH-Rural Hospitals, AAMs- Ayushman Arogya Mandir.

† Refers to facilities providing medical and/or surgical abortion services in the study block or district.

### Study population

The study will include two key participant groups: married women aged 18–49 years who have availed or potential or current users of abortion services, and health system stakeholders responsible for or engaged in the delivery of Comprehensive Abortion Care. For the Maharashtra study site, eligibility will also extend to women in live-in arrangements that are socially recognized within local customary practices, and who self-identify as married, acknowledging the contextual specificity of marital norms in this setting.

#### Quantitative component.

Married women aged 18–49 years residing in the selected study block will be selected for quantitative household survey. In addition, all public health facilities within the study block (like: Community Health Centres (CHCs), Primary Health Centres (PHCs), Rural Hospitals, and Sub-District Hospitals (SDHs)) will be included for facility assessments.

#### Qualitative component.

For the qualitative data collection, married women aged 18–49 years who have experienced an abortion within the preceding two years (identified from the household survey) will be purposively selected to explore their experiences in availing abortion services. Additionally, key stakeholders across the health system will be included to understand facilitators and barriers to abortion service delivery. These will include healthcare professionals such as Community Health Officers (CHOs), Auxiliary Nurse Midwives (ANMs), Gynaecologists, Medical Officers, Staff Nurses, District Health Authorities, Facility Managers, and community health workers including Accredited Social Health Activists (ASHAs) and Anganwadi Workers (AWWs).

### Sample size and sampling

#### Quantitative sample size.

Six MRHRUs were purposively selected to represent diverse geographic regions of the country. For the community-based survey, the sample size was calculated assuming a 50% prevalence of awareness of safe abortion services due to the absence of relevant Indian literature, with a 95% confidence level and 5% absolute precision. This yielded a base sample size of approximately 384 married women. After accounting for a conservatively assumed design effect of 2, specified a priory for sample size planning, and a 10% non-response rate, the final target sample size was estimated at 854 married women aged 18–49 years per site. As abortion is a sensitive subject, We are conducting a household based survey, some eligible participants may decline participation or may not feel comfortable responding during household-based interviews. To address this possibility and ensure adequate sample size, a conservative non-response rate of 10% was considered. A multistage cluster sampling design will be used. The sampling frame will be prepared using Accredited Social Health Activist (ASHA) lists of married women aged 18–49 years, which will serve as the measure of size (MOS) for each village. Based on the calculated total sample size and the decision to conduct the study across 20 clusters, it was determined that 42 interviews would need to be conducted per cluster to achieve the required overall sample size.

In the first stage: 20 villages (clusters) will be selected using probability proportional to size (PPS) sampling, employing a random start and fixed sampling interval. For, MRHRU-Khumulwng (Tripura), hamlets will be considered as cluster, as the hamlets serves as independent settlement units. In the second stage: a complete household listing will be undertaken in each village to identify household with at least one eligible women. A systematic sampling technique will be used to select 43 eligible women per village using a random start and fixed interval. Households will be approached using the right-hand rule, and when more than one eligible woman present, one will be selected using the Kish grid or next birthday method [18]. In villages with fewer than required number of sample, all will be included and the remaining sample will be drawn from the nearest village/hamlet or an adjacent village within the same Gram Panchayat.

For the facility assessment survey, a total enumeration sampling strategy will be applied, whereby all public health facilities located within the study blocks will be included.

#### Qualitative sampling.

Participants for the qualitative data collection will be selected using purposive sampling with a maximum variation approach to ensure representation across facility types, provider cadres, and geographic contexts. The sample will include healthcare providers and women aged 18 –49 years who have either accessed abortion services from the public health facilities or availed abortion care elsewhere within the past two years. At each MRHRU site, approximately 25 healthcare providers and 15 care recipients or seekers will be sampled. Sampling will be guided by the principle of thematic saturation [18,19] within individual sites and across six MRHRU sites representing different regions. Overall, approximately 232–240 qualitative interviews are proposed, with final sample size guided by thematic saturation and contextual requirements across sites. In addition, 18 focus-group-discussion (three per site) comprising Accredited Social Health Activists (ASHA) and Anganwadi Workers (AWW) will be conducted to capture frontline health worker’s perspectives. This recruitment method will facilitate varied samples and allow for adaptability. The detailed distribution of the qualitative sample is provided in Table 2.

**Table 2:** Sample size for qualitative data collection across six MRHRU Sites.

S.N	MRHRU	Health Administration		Abortion Care Provider				Facility manager/Incharge	Abortion Care Recipient /Seeker		Total interviews per MRHU
		CMO	Dy. CMO/DPM/DAC	Obstetricians/Gynaecologist	SMO /MO	Staff Nurse	CHO/ANM		Exit interview with women availed CAC services from public health facility	Women who had Abortion	
	Tigiria (Cuttack, Orissa)	1	1	2-3	3-5	3-5	3-5	3-5	3-5	8-10	**32-40**
	Namkum (Ranchi, Jharkhand)	1	1	2-3	3-5	3-5	3-5	3-5	3-5	8-10	**32-40**
	Dahanu (Palghar, Maharashtra)	1	1	2-3	3-5	3-5	3-5	3-5	3-5	8-10	**32-40**
	Tripura Khumulwng	1	1	2-3	3-5	3-5	3-5	3-5	3-5	8-10	**32-40**
	Pali, Uttar Pradesh	1	1	2-3	3-5	3-5	3-5	3-5	3-5	8-10	**32-40**
	Raichur Karnataka	1	1	2-3	3-5	3-5	3-5	3-5	3-5	8-10	**32-40**
**Total**		**6**	**6**	**18**	**30**	**30**	**30**	**30**	**30**	**60**	**192-240**

### Data collection methods

#### Quantitative data.

Before collecting data, all the study participants will be informed about the purpose of the study and written informed consent will be obtained. The research instruments will be administered by trained field staff across all sites. A structured household survey questionnaire will be used to collect information on socio-demographic characteristics (such as age, residence, education, occupation, socio-economic status), reproductive history (i.e., gravida, parity, number of living child and, age of living child), pregnancy outcomes (such as: spontaneous abortion, induced abortion, stillbirth), knowledge and awareness of abortion services and options, utilization of abortion services, knowledge about danger signs, post abortion care, contraceptive use, and barriers faced in accessing care.

The questionnaire underwent a multi-step development and validation process, including internal review by the Principal Investigators of each study site, external expert review by national-level public health and qualitative research specialists, and validation through a one-day in-person workshop. Following these reviews, the tools were pilot-tested in the field to assess clarity, comprehensibility, and contextual relevance. Feedback from piloting was incorporated, and final refinements were made to ensure cultural and linguistic appropriateness across all study sites. The finalized tools were then translated into local languages using forward and back-translation to maintain conceptual equivalence and consistency. All quantitative data will be captured electronically using KoboCollect and stored on a secure, password-protected cloud server with restricted access, in accordance with data protection and confidentiality protocols.

#### Qualitative data.

The qualitative data will be collected through Key informant interviews (KIIs), in-depth interviews (IDIs), and focus group discussions (FGDs). Along with this, observation method will be used to document the roll out of the Comprehensive Abortion Care (CAC) guideline at facility level. Separate semi-structured interview guides for each category of service providers have been developed using a staged process informed by the Comprehensive Abortion Care (CAC) Guidelines (2023). The guides covered components such as provider roles, service availability, referral mechanisms, post-abortion care, community responses, barriers to access, and service improvement. Along with KIIs, a separate Focus Group Discussions (FGDs) guide (comprising knowledge and awareness about abortion, prevalent abortion care practices and the social norms and stigma in the community) has been prepared to collect data from the Accredited Social Health Activists (ASHAs) and Anganwadi workers who act as the community health worker and are responsible for providing counselling and referral to health care facilities. The study tools were developed using a systematic and rigorous approach informed by an extensive literature review, consultations with national-level experts in the subject area, and qualitative research. Key literature findings that informed guide development included decision-making processes, information pathways, care-seeking trajectories, quality of care, stigma, and other contextual barriers and facilitators influencing access and experiences of care. The CFIR framework also informed the development of the guides to ensure that relevant implementation domains and contextual factors were adequately captured. All tools underwent internal review, external expert review, validation through an in-person workshop, piloting, and contextual refinement. Guides were translated into local languages using forward and back-translation. Pretesting of the tool was done in a separate block.

Trained researchers (preferably female) will collect qualitative data with minimum graduate and post graduate degree in social science, or public health or related discipline, and demonstrated experience in qualitative research and sensitive interviewing. Prior experience in community based data collection will be considered during recruitment. A multi-level capacity-building approach will be adopted for the selection and deployment of qualitative data collection teams across study sites. In the first phase, investigators and study coordinators from all participating sites will undergo orientation and capacity building workshop. Subsequently, the trained personnel will recruit the research team at their respective sites.

Following recruitment, a comprehensive two-day in-person training workshop will be conducted for the research teams from all sites at a central location. The teams will be trained in qualitative research methods, interviewing techniques, ethical conduct, informed consent, confidentiality, non-judgmental interviewing techniques, probing techniques, and management of sensitive conversations and participant distress. Training will be delivered by a multidisciplinary team with significant expertise in sensitive research areas, including reproductive health and maternal mental health, with backgrounds in community medicine, social anthropology, and ethnographic research. Following initial training, the research team will continue to receive regular orientation, mentoring, and support through reflective debriefing sessions conducted both online and onsite throughout the data collection period to strengthen consistency, address field challenges, and enhance reflexive practice.

The interviewer will be familiar with the local language and socio-cultural context but will have no prior relationship with study participants before or during the data collection process, thereby minimizing potential bias and facilitating independent and ethically appropriate engagement.

To ensure participants’ full participation, comfort, privacy and confidentiality, only the study team and respective participants will be present at the time of data collection. Presence of non-participants will be avoided during the interview unless it seems essential due to the safety of the researcher or the researched. Interviews and FGDs will be conducted in private settings and at the place of participant’s choice to minimize any reputational risk. In cases where women may not be able to complete the interview due to privacy or any other reasons and wishes to continue later, repeat interviews will be carried out as per their convenience. Written or verbal informed consent will be obtained. Discussions will be audio-recorded with permission; otherwise, detailed field notes will be maintained. Reflexive field notes will be documented throughout data collection.

All interviews will range between 45–60 minutes on average depending on willingness and availability of the participants and will be audio recorded after obtaining either a written or verbal consent, depending on the comfort and willingness of the participant. Participants across study groups will be provided a participant information sheet in both English and the regional language. Throughout the data collection and analysis period, the research team will be engaged in bracketing exercise to reflect on and acknowledge their own assumptions, prior knowledge, values, and positionalities on issues such as abortion and abortion care. Qualitative data collection and analysis will occur concurrently, allowing iterative reflection on emerging findings and enabling refinement of data collection processes and discussion guides where necessary throughout the study period. An audit trail will be maintained throughout the research process to systematically document the entire research process (including the decisions related to research design, tool preparation, sampling, data collection, coding and interpretation) to enhance the credibility and transparency of the findings. Similarly, regular debriefing and reflective discussions will be conducted throughout data collection to refine discussion guides where necessary. All modifications in the study tool will also be documented in the audit trail.

### Data analysis

#### Quantitative analysis.

Quantitative data from facility assessments and household surveys will be retrieved from the data management system and analysed using statistical software like STATA (or an equivalent statistical software). Initially, data cleaning and validation will be done using built-in system checks and manual verification. Data will be analysed at both an overall and site-specific level to observe the variation within and across study sites in knowledge, awareness, experiences, and attitudes related to CAC services. In addition to identifying the common barriers and facilitators, the analysis will also explore how these factors vary across study sites and influence service delivery and utilization within different local contexts. Descriptive statistics will be used to summarize sociodemographic characteristics, reproductive history, and service-related variables. Continuous variables will be reported as means with standard deviations (SD) or medians with interquartile ranges (IQR), as appropriate. Categorical variables will be reported as frequencies and percentages. The study will use survey design commands and appropriate sample weight to account for village-level clustering. Prevalence estimates will be reported with 95% confidence intervals (CIs).

A composite facility readiness index will be constructed a priori using predefined indicators derived from the Comprehensive Abortion Care (CAC) Guidelines (2023) and the Comprehensive Abortion Care: A Health Facility Assessment Tool (2023) by WHO. Each indicator will be assessed through direct observation or facility records and coded as binary (available and functional on the day of assessment = 1; not available or non-functional = 0), and aggregated to generate both domain-specific scores and an overall readiness classification. Facilities meeting all minimum readiness criteria will be classified as “ready”, if they demonstrate availability and functionality on the day of assessment for the following minimum criteria: 1. Minimum one CAC trained provider; 2. Availability of the essential drugs and consumables; 3. Functioning equipment for the surgical method of abortion; 4. Availability of proper method of abortion in the facility; 5. A functional referral system. A facility lacking any of these essential components will be considered as “non-ready”. The proportion of ready facilities will be estimated with 95% CIs and compared across facility types using appropriate statistical tests.

Provider and women’s knowledge scores will be computed as summative indices, with each correctly answered item assigned a score of 1 and incorrect or “don’t know” responses assigned a score of 0. Item scores will be summed to generate total knowledge scores. Facility-based abortion utilization and post-abortion contraceptive uptake will be analyzed as binary outcomes.

Missing data will be assessed for each variable. For the primary outcome, complete case analysis will be used if missingness is below 5%; otherwise, multiple imputation by chained equations (MICE) will be performed under the missing at random assumption.

Quantitative findings will inform interpretation and integration with qualitative results during mixed-methods analysis.

#### Qualitative analysis.

The methodological orientation of the study will include a combined analysis using phenomenology and thematic analysis which allows for a systematic identification and interpretation of patterns across the study sites and stakeholder groups (providers, administrators, community actors, and women). All the interviews will be first transcribed verbatim by the same interviewer in the language in which the interview was conducted following the process of de-identification (by removing name, address and any other information revealing the identity). In the second stage, the transcripts from all regional languages will be professionally translated to English. In the third stage, translated interviews will be coded and analysed using qualitative data management software (NVivo or equivalent softwares). A combination of deductive and inductive thematic analysis will be employed to generate comprehensive insights. An initial broad coding framework will be developed to guide analysis across sites and participant groups and will be iteratively refined throughout the analytic process to incorporate emerging themes and contextual variations. The preparation of the deductive codebook will be guided by the Consolidated Framework for Implementation Research (CFIR), which offers a structured lens to explore multilevel determinants of implementation. Specifically, codes will be drawn from CFIR domains including Intervention (CAC interventions) characteristics, inner setting (intervention deliverers), outer setting (in which inner setting is lying), characteristics of Individuals (recipient and deliverers of the interventions), and process (implementation of intervention), capturing aspects such as perceived complexity of CAC service delivery, organizational culture, external policy influences, provider knowledge and attitudes, and the role of training and engagement. This approach will ensure systematic examination of implementation barriers and facilitators. In parallel, inductive codes will be developed by two lead researchers who would first read and reread transcripts to gain familiarity with the data following which they will code two transcripts from each site and prepare the inductive codebook after discussing any discrepancies, collapse codes and adding any new codes based on emergent themes. The entire research team involved in analysis will be oriented about the definition of each code in the revised codebook for consistent application across all the transcripts. In the last stage, all the emerged themes will be analysed to identify similar or different patterns across the sites. Following this, thematic analysis will be conducted to interpret meanings and relationships relevant to all four research objectives. Throughout this process, the coding and analytic decisions will be checked for consistency, and representative quotations will be identified to support key themes in the findings for a transparent and rigorous analysis of the qualitative data. To address site specific issues, the analysis will examine how common barriers and facilitators operate across different contexts, with particular attention to site-specific variations that may influence implementation processes and outcomes. Similarly, variations across participant groups and sites will be examined using three data sources, including data from different participant groups, contextual information, and field observations where necessary.

#### Integration and model development.

Findings from the quantitative and qualitative components will be triangulated to generate a comprehensive understanding of the current status of CAC services and their determinants. Converging evidence will be used to inform the development of a preliminary implementation framework (“Model 0 + ”), rooted in national CAC guidelines but refined based on empirical insights. The Model “0+” will map identified barriers (e.g., supply gaps, provider training, and stigma) and facilitators (e.g., community engagement, referral efficiency) to potential strategies. This model will be shared with stakeholders in a consultative workshop, allowing for validation and refinement based on field realities and policy relevance. The finalized model will be co-designed involving the research team and government stakeholders.

### Data management

All data will be managed centrally at the coordinating site. The site will utilize KoboCollect platform which is widely used for health systems research. This platforms will support real-time data capture, secure storage, role-based access, and advanced data validation functions, thus enhancing data quality and efficiency. Each MRHRU site will have field-based data entry staff or investigators using electronic tablets equipped with custom-designed forms. Data from household surveys, and facility interviews will be uploaded daily to the central server, either through mobile internet or periodic sync when offline. Additional logical consistency and validation checks will be conducted at the coordinating site. Queries will be generated and shared with field teams for clarification and correction. Cleaned and verified datasets will be archived. The quality assurance outputs will be reviewed during regular data review calls and debriefing sessions with the field teams to ensure completeness and accuracy.

### Data security

All personal data will be handled in compliance with the Indian Council of Medical Research Data Protection Regulation. All data obtained during the course of the study will be kept confidential, and all project team members will be bound by data secrecy. All data will be anonymized and retained in compliance with the relevant data protection regulations. The quantitative survey data will be anonymized before statistical analysis. All qualitative data will be analyzed in De-identified or pseudonymized form, ensuring the names of individuals, organizations, or places will not be publicly available. Recorded data will be removed at the request of participants as long as it has not yet been anonymized.

### Study status

This study is planned to begin on August’2025, and finish on July’2026. Quantitative and qualitative data collection is set to be completed between February’2026, and May’2026. The data collection was initiated in a phased manner across study sites depending on ethical and administrative approval. Site-wise data collection details were provided in supplementary table S1 in S1 File.

### Ethics and dissemination

This study involves sensitive topic (abortion) and human participants. This protocol has been reviewed and approved by the Institutional Ethics Committees from each MRHRU site (ICMR institute) viz. Institutional Human Ethics Committee, ICMR – Regional Medical Research Centre, Bhubaneswar (Ref: 2025/IHEC/M1/01, dated: 07.02.2025) Written informed consent will be obtained from all participants. Each participant will have the right to decline or terminate their participation at any point before or during data collection, with or without justification. Participants will be assured that refusal to participate will not affect any services they receive at the government health facilities. In order to guarantee complete anonymization, numerical identifiers will be utilized instead of names for all data. Research outcomes will be formally disseminated through peer review and conference proceedings.

## Discussion

India has invested considerable effort over the years to strengthen access to safe abortion through a supportive legal framework and detailed national guidelines on CAC. Ideally, these provisions should ensure that women are able to access safe and affordable abortion services within the public health system. However, whether these policies are being implemented as intended, especially in rural and underserved areas, is far less clear. This study has been designed to interrogate this implementation gap by examining how CAC services are organized, delivered, and experienced within routine public-sector settings in rural India. Access to abortion care is not determined by policy alone. Even where services are officially available, women’s decisions to seek care from public facilities are shaped by multiple factors, including awareness of legal entitlements, perceptions of quality and confidentiality, fear of stigma, and social influences within families and communities. At the same time, providers operate within health systems that may be constrained by staff shortages, limited training opportunities, supply gaps, and competing service priorities. By bringing together perspectives from women, providers, and facilities, this study adopts a systems-oriented lens that moves beyond single level explanations. A key strength of the proposed study is its focus on rural and tribal settings across different regions of the country, using Model Rural Health Research Units (MRHRUs) as platforms. Rural areas continue to account for a large share of unsafe abortions [21], yet there is relatively little empirical evidence on how CAC services are organized and delivered in these settings. Conducting the study across multiple MRHRUs will allow comparison across diverse contexts and help identify which challenges are common across settings and which are more context-specific. The diversity of geographic, socio-cultural, and health system contexts represented in this study allows identification of both common bottlenecks and context-specific challenges, thereby informing strategies that are adaptable rather than one-size-fits-all. Such insights are essential for developing strategies that are realistic, locally appropriate, and potentially scalable. The mixed-methods design is particularly important for a topic like abortion care. Quantitative data can show where services exist and how often they are used, but they cannot fully explain why women do or do not access these services, or why providers may struggle to offer them consistently. Qualitative data will help unpack these issues, including the role of stigma, provider attitudes, informal care pathways, and health system constraints. Integrating these findings will allow the study to generate nuanced insights that are directly relevant for strengthening service delivery. This study represents the first phase of a larger, multi-phase implementation research programme. Rather than assuming shortcomings in the national CAC guidelines, the study treats them as a starting point (“Model 0”) and seeks to understand how they play out in routine practice. The proposed development of a refined implementation strategy (“Model 0+”) is intended to be grounded in real-world evidence and informed by the voices of women and health-care providers. Anchoring qualitative analysis within the Consolidated Framework for Implementation Research (CFIR) further strengthens interpretation by situating findings within a well-established implementation science framework, thereby increasing feasibility, acceptability, and potential for scale-up in subsequent phases. However, certain limitations are anticipated. The focus on married women may limit insights into the experiences of unmarried women and women aged 15–18 years, who may face heightened stigma and distinct social, cultural and health sector barrier to access CAC services. Consequently, the findings may leads to limited generalizability to these populations. Additionally, findings will also be specific to the selected MRHRU sites and may not be fully representative of all rural settings in India. However, as an implementation-focused study, the goal is not national prevalence estimation but generation of transferable lessons on service delivery processes and system constraints. Future research should focus on the experiences, barriers, stigma, and care-seeking pathways of unmarried women and women aged 15–18 seeking abortion services in rural India, in an effort to generate evidence that can inform more inclusive and responsive Comprehensive Abortion Care programs and policies. By identifying both gaps and enabling factors across the health system, the findings are expected to inform practical strategies to strengthen CAC implementation, improve women’s access to safe abortion services, and support ongoing efforts to translate policy commitments into meaningful improvements in service delivery and access to care.

## Supporting information

S1 File Site-wise data collection details were provided in supplementary table titled ‘Site-wise data collection status’ provided in supplementary file. Supplementary Material 1: GRAMMS checklist.
(DOCX)

S2 File Supplementary Material 2: Study Tools. (ZIP)
