# Supplementary material for: Status, barriers and facilitators of comprehensive abortion care services in rural India: A multicenter mixed-methods study protocol
Source: PLoS One. 2026 Sep 16;21(9):e0358274. doi: 10.1371/journal.pone.0358274 (PMC13581207; doi:10.1371/journal.pone.0358274)
Supplement: Supplementary file 1 [file pone.0358274.s001.docx]

**Supplementary Table 1: Site-wise data collection status**

| **Study Site** | **State** | **Data Collection Start Date** | **Status** |
| --- | --- | --- | --- |
| MRHRU Tigiria | Odisha | 20^th^ Jan’2026 | Ongoing |
| MRHRU Namkum | Jharkhand | 20^th^ Jan’2026 | Ongoing |
| MRHRU Dahanu | Maharashtra | 21^st^ Jan’2026 | Ongoing |
| MRHRU Khumulwng | Tripura | 19^th^ Jan’2026 | Ongoing |
| MRHRU Pali | Uttar Pradesh | 23^rd^ Jan’2026 | Ongoing |
| MRHRU Raichur | Karnataka | 22^nd^ Jan’2026 | Ongoing |

**Supplementary Table 2: Good Reporting of A Mixed Methods Study (GRAMMS) checklist**

**Title:** Status, Barriers and Facilitators of Comprehensive Abortion Care Services in rural India: A Multi-centric mixed methods study

| **Guideline** | **Section: page** |
| --- | --- |
| Describe the justification for using a mixed methods approach to the research question | Methods- under Study design, page 5-6 |
| Describe the design in terms of the purpose, priority and sequence of methods | Methods- under Study design, page 5-6 |
| Describe each method in terms of sampling, data collection and analysis | Methods- under Sample and study setting, Page 6-11  Methods- under Data collection, Page 11-13  Methods- under Data Analysis, Page 14 and 16 |
| Describe where integration has occurred, how it has occurred and who has participated in it | Methods- under Integration and Model Development, Page 16 |
| Describe any limitation of one method associated with the present of the other method | Discussion, Page 18-19 |
| Describe any insights gained from mixing or integrating methods | Discussion, Page 18-19 |

*Ref: O'Cathain A, Murphy E, Nicholl J. The quality of mixed methods studies in health services research. J Health Serv Res Policy. 2008;13: 92-98*
