# Supplementary material for: Status, barriers and facilitators of comprehensive abortion care services in rural India: A multicenter mixed-methods study protocol
Source: PLoS One. 2026 Sep 16;21(9):e0358274. doi: 10.1371/journal.pone.0358274 (PMC13581207; doi:10.1371/journal.pone.0358274)
Supplement: Supplementary file 2 [file pone.0358274.s003.docx]

Dear Editor,

We sincerely thank the Editor and Reviewers for their careful evaluation of our manuscript and for their constructive and insightful comments. We have revised the manuscript accordingly and believe that these changes have strengthened the clarity and quality of our work.

In the table below, we provide a point-by-point response to each comment. All changes have been incorporated in the revised version.

| **Reviewer** | **Editorial comments** | **Response** | **Page and line number** |
| --- | --- | --- | --- |
| 1 | You state that "Quantitative and qualitative data collection is set to be completed between February’2026, and May’2026. | Thank you for this query. Data collection for the study is currently ongoing and has not yet been completed. As per the revised study timeline, quantitative and qualitative data collection is expected to be completed by May 2026. We have updated the manuscript to clearly reflect the current status of data collection and the expected completion timeline. | Page no: 398-402 |
|  | You state that "This study is planned to Please can you specify the data collection start date for each of the study sites | Thank you for this suggestion. Data collection across the study sites is being initiated in a phased manner, depending on the completion of site-specific administrative and ethical approvals. In general, data collection across sites commenced from February 2026, with slight variations between sites. We have now clarified this in the manuscript by specifying that data collection is being conducted in a staggered manner across sites, along with the overall timeline. | Page no: 398-402 and Supplementary Table 1 |
