# Supplementary material for: Status, barriers and facilitators of comprehensive abortion care services in rural India: A multicenter mixed-methods study protocol
Source: PLoS One. 2026 Sep 16;21(9):e0358274. doi: 10.1371/journal.pone.0358274 (PMC13581207; doi:10.1371/journal.pone.0358274)
Supplement: Supplementary file 3 [file pone.0358274.s004.docx]

1. In this instance it seems there may be acceptable restrictions in place that prevent the public sharing of your minimal data. However, in line with our goal of ensuring long-term data availability to all interested researchers, PLOS’ Data Policy states that authors cannot be the sole named individuals responsible for ensuring data access (http://journals.plos.org/plosone/s/data-availability#loc-acceptable-data-sharing-methods).

Data requests to a non-author institutional point of contact, such as a data access or ethics committee, helps guarantee long term stability and availability of data. Providing interested researchers with a durable point of contact ensures data will be accessible even if an author changes email addresses, institutions, or becomes unavailable to answer requests.

Before we proceed with your manuscript, please also provide non-author contact information (phone/email/hyperlink) for a data access committee, ethics committee, or other institutional body to which data requests may be sent. If no institutional body is available to respond to requests for your minimal data, please consider if there any institutional representatives who did not collaborate in the study, and are not listed as authors on the manuscript, who would be able to hold the data and respond to external requests for data access? If so, please provide their contact information (i.e., email address). Please also provide details on how you will ensure persistent or long-term data storage and availability.

**Response:** Thank you for this clarification.

The study data will be stored electronically at the Indian Council of Medical Research (ICMR) Headquarters, New Delhi, and retained in accordance with institutional data management and record-retention policies. Given the sensitive nature of the data to be collected, including information related to abortion care services, only de-identified datasets will be made available to qualified researchers upon reasonable request and subject to applicable ethical and confidentiality requirements.

To ensure long-term accessibility independent of the study authors, requests for data access may be directed to the Nodal Officer for Data Management, ICMR Headquarters, who is not involved in the study and is not listed as an author on the manuscript. The contact details of the Nodal Officer have been provided below. The study data will be maintained by ICMR Headquarters under institutional oversight to ensure persistent storage and long-term availability beyond the duration of the study and independent of the study investigators.

The contact details of the Nodal Officer are as follows:

Ajay Singh Dhama

Scientist-C

Indian Council of Medical Research, New Delhi

Email: [ajaysinghdhama.hq@icmr.gov.in](mailto:ajaysinghdhama.hq@icmr.gov.in)
