# Supplementary material for: Status, barriers and facilitators of comprehensive abortion care services in rural India: A multicenter mixed-methods study protocol
Source: PLoS One. 2026 Sep 16;21(9):e0358274. doi: 10.1371/journal.pone.0358274 (PMC13581207; doi:10.1371/journal.pone.0358274)
Supplement: Supplementary file 4 [file pone.0358274.s005.docx]

# **Response to Editor**

**Additional Editor Comments:**

All concerns of the reviewers have been addressed satisfactorily sans one comment of the editor.

The design effect is calculated using the formula, assuming equal cluster sizes: 1+(m-1)ρ where m= average number of individuals sampled per cluster. ρ= intracluster correlation coefficient. In case of unequal cluster sizes, following formula is used: design effect=1+[m(1+CV*2)-1]ρ

An arbitrary design effect is statistically not tenable.

I understand that the project has been approved by ICMR and modifications are not possible. Nonetheless, for the sake of the readers of the journal, it is pertinent to mention how the effect size was determined.

Authors may consider explaining that the design effect was arbitrary.

**Response**

We thank the Editor for this important methodological observation. We agree that the design effect of 2 used in the protocol was not derived empirically from an estimated intracluster correlation coefficient (ICC) and average cluster size. Rather, it was specified a priori as a conservative planning assumption for the sample-size calculation, given the cluster-based study design and the absence of reliable prior ICC estimates for the study setting and outcome at the time of protocol development.

We have now clarified this in the manuscript to ensure transparency for readers. We have also explicitly stated that the design effect of 2 was a conservative a priori assumption used for sample-size planning, rather than an empirically estimated parameter.

Line and Page no: Page no 10; Line no: 198-199
