# Supplementary material for: Status, barriers and facilitators of comprehensive abortion care services in rural India: A multicenter mixed-methods study protocol
Source: PLoS One. 2026 Sep 16;21(9):e0358274. doi: 10.1371/journal.pone.0358274 (PMC13581207; doi:10.1371/journal.pone.0358274)
Supplement: Supplementary file 5 [file pone.0358274.s002.zip › CAC KII_Guide for CMO.docx]

**In-depth Interview Guide for Health Care Professionals**

**[Chief Medical Officer (CMO)/Chief District Medical Officer (CDMO)]**

**Study Title:** Status, Barriers, and Facilitators of Comprehensive Abortion Care Services: An Model Rural Health Research Units (MRHRU) Based Study

**Objective:** To understand how senior health care professionals (Chief Medical Officer) provide and support Comprehensive Abortion Care (CAC) services, focusing on quality assurance through training and capacity building, service delivery and supply chain management and Monitoring, Reporting of CAC services.

**Introduction**

Thank you for taking the time to speak with me today. My name is [interviewer name], and I am part of the Indian Council of Medical Research (ICMR) team. Before we begin, can I please confirm that you have received a copy of the study information sheet and consent form?

As a reminder, this project aims to understand the circumstances, decisions, care-seeking behaviours, and experiences related to abortion. We are conducting this study to identify the factors that influence how people access abortion-related care, the quality of care received, any challenges faced, and how the health care provision can be improved to better support individuals in similar situations. Our goal is to learn from your experience to strengthen the healthcare system and ensure people have access to safe, respectful, and appropriate care.

We want to understand things from your perspective and every response is valuable for this study. Everything you share with us will be treated with full discretion. Your responses will be kept confidential and only be used for research purposes. We also assure you that your identity will not be linked to any of the findings. The interview will take approximately 45 minutes, depending on how much you would like to share. May I check if you are free to speak for that amount of time now?

I would also like to request your permission to audio record our conversation. This will help me capture your responses accurately and focus on listening to you rather than taking detailed notes. Is it okay for me to start recording now?

Thank you.

I**nformation to collect (before starting):**

| **Name** |  |
| --- | --- |
| **Age (in years)** |  |
| **Education Qualification** |  |
| **Year of Experience** |  |
| **District** |  |
| **Date of the interview** |  |

### A. ****Rapport-Building / General Questions****

1. Could you briefly tell about your role and responsibilities as the Chief Medical Officer (CMO) in this district?

*[How long it’s been since have you taken the charge as the CMO; Administrative, clinical, and supervisory work? programs or initiatives directly overseeing?]*

2. What are some of the major public health priorities you are currently focusing on in the district?

*[Antenatal care & institutional deliveries; Immunization coverage & child health programs; Contraception & safe abortion services; TB, malaria, HIV prevention programs; Nutrition supplementation & anaemia control; Staffing, training, and deployment issues; Facility upgrades & equipment availability; Health data collection & reporting system; Community awareness & outreach activities; Implementation of government schemes]*

3. From your experience, what are the major challenges in strengthening maternal and reproductive health services here?

*[Staffing shortages, high workload, Inadequate training or skill gaps, Facility infrastructure limitations, Drug, equipment, supply shortages, Community awareness, health-seeking behaviour, Cultural or social barriers, Funding or resource constraints, Data collection and reporting challenges, Referral system inefficiencies, Coordination with NGOs, private sector]*

4. From your experience, what factors or strategies have helped improve maternal and reproductive health services in the district, and how have you managed to address these challenges?

**CAC Related questions**

B. **Administrative and Regulatory Oversight**
5. **Are CAC services provided in the district both by public and private facility?**
6. **How much time it takes from application to approval of a facility for provision of MTP services (please verify timeline between application form and their approval)?**
7. How do you ensure that abortion services in your district comply with the MTP Act and CAC guidelines?

*[Monitoring and checking facility registration, approvals and legal compliance; Provider training, certification, skill verification; Record-keeping, documentation, reporting systems; Monitoring, inspections, supervision mechanisms; Handling legal or ethical issues]*

8. What challenges do you face in approving and monitoring facilities that provide CAC services at public health facilities? *[Human Resource, lack of training, lack of infrastructure, ]*
9. What challenges do you face in approving and monitoring facilities that provide CAC services at public health facilities? [Please specify]
C. **Service Delivery Management**
10. How do you ensure that CAC services are available and accessible across PHCs, CHCs, and other facilities? [IEC/BCC, SBCC through Capacity building, through community health workers]
11. Does the health facility in the district displace citizen charter conveying availability of safe abortion services?
12. What mechanisms are in place to strengthen referral systems for complicated CAC cases?

*[Structured referral protocols and guidelines; Communication between PHCs, CHCs, and other facilities; Transport and ambulance availability; Tracking and follow-up of referred cases]*

13. How do you deal with extraordinary cases of? Do you have a medical board to monitor and support such cases?

[*Cases of Abortion post 24 week, Abortion in case of a minor or rape victim; any such case that you can share?]*

D. **Quality Assurance and Capacity Building**
14. Are the service providers at public health facilities trained and oriented on maintaining dignity, privacy and confidentiality? (to be verified through exit interview and available facility infrastructure)
15. In your view, what are the key gaps in maintaining quality of care such as privacy, counselling, and informed consent in abortion services?

*[Lack of training and awareness in care providers; lack of adequate infrastructure]*

16. What strategies have you adopted to train and support providers (medical officers, nurses, ANMs) in CAC?

*[Training programs; hands-on practice; Supportive supervision; Guidelines, protocols, and reference materials; Incentives, recognition, or motivation strategies]*

E. **Supply Chain and Resource Management**
17. How do you monitor the availability of essential drugs (mifepristone, misoprostol etc.) and equipment for CAC services in your district?

[*Reporting systems for shortages or stock-outs; Supervision of facility-level supply management]*

18. What are the major barriers you encounter in ensuring uninterrupted supplies and how do you address them?

[*Procurement delays; supply chain issues; Stock-outs at PHCs, CHCs, hospitals; Funding or budget constraints]*

F. **Monitoring, Reporting, and Coordination**
19. Could you describe how cases of abortion are documented in your district?

*[Facility Registers, MTP forms, Entry on any portal]*

20. How do you coordinate with other stakeholders (state health authorities, NGOs, or private facilities) to improve CAC services?

[*Ensuring regular CAC reporting, Conducting meetings, Partnerships with NGOs]*

21. In your observation, at which level of the health system (PHC, CHC, SDH, or DH) are CAC services still inadequate, and what do you think are the main reasons for these gaps?

*[*Probes: *Shortage of trained staff, overload of patients, infrastructure limitations, training gaps, supply issues, referral challenges, community barriers, or administrative constraints.]*

22. From your observation, how supportive are the doctors, nurses, and other staff in prioritizing CAC services compared to other health services in your district?

*[Probe: Prioritize CAC services, follow CAC guidelines]*

G. **Initiatives**
23. What steps have you taken to address social stigma or misconceptions around abortion services in the community?

[*Organizing training, Community awareness campaigns, emphasis on Counselling services, Collaboration with NGOs or outreach programs, Media or communication strategies to reduce stigma, Ensuring availability and distribution of IEC materials*.]

24. In the last, what one change do you think, would make it easier for women to access safe abortion care at the facilities in your area?

**Closing Remark**

Thank you very much for your time and for sharing your experiences. Your input is very important and will help improve reproductive health services for women in your community.

What steps have you taken for the wider dissemination of CAC services in your district?
