# Supplementary material for: Status, barriers and facilitators of comprehensive abortion care services in rural India: A multicenter mixed-methods study protocol
Source: PLoS One. 2026 Sep 16;21(9):e0358274. doi: 10.1371/journal.pone.0358274 (PMC13581207; doi:10.1371/journal.pone.0358274)
Supplement: Supplementary file 5 [file pone.0358274.s002.zip › CAC FGD Fuide for ASHA.docx]

**Focus Group Discussion (FGD) Guide for ASHA/AWW WORKERS**

**Study Title:** Status, Barriers, and Facilitators of Comprehensive Abortion Care Services: An Model Rural Health Research Units (MRHRU) Based Study

**Objective:** To explore ASHA workers’ experiences, perceptions, and challenges in promoting and supporting Comprehensive Abortion Care (CAC) services within their communities, including counselling, referrals, and addressing stigma.

**Targeted Participants**

8–10 Community Level Health Workers [e.g., Accredited Social Health Activist (ASHAs), Anganwadi workers (AWW)] working in the region in focus.

**Duration**

An ideal Focus Group Discussion (FGD) should be carried out following a duration between 60–90 minutes, depending on the number of participants and the quality of discussion.

**Team**

The team conducting the FGD should include one Moderator and one Note-taker for better facilitation and quality control of the discussion.

**Language**

The moderation of the discussion may be conducted by a non-native speaker; therefore, a native/local person should be there to support with instant translation of local terms, expressions, and culture specific references. However, moderator should prioritize local language as mode of the discussion for participant’s comfort. The native speaker must assist in interpreting both the participant’s response and the moderator’s questions as needed to maintain clarity and accuracy in communication.

**Notes for the team conducting the FGD**

- *While conducting the FGD, observe and note the leading participants, and encourage those too who are quiet or speaking less during discussion.*
- *Along with the verbal comments or remarks, give close attention to the non-verbal cues such as pause, silence, discomfort, or hesitation.,*
- *Ensure to show respect when discussion is around sensitive topics.*
- *Pause and provide a moment of silence after each question to allow the participants for reflection or any other comments/ remarks from them.*

**Introduction**

Thank you for joining today’s discussion. We have invited you all in this discussion for an Indian Council of Medical Research (ICMR) led research project in which we are trying to understand what abortion related services and facilities are available at different health centres and whether these services are working properly. Part of this discussion would also focus on your experiences and the challenges you come across while helping women access abortion-related care. Based on what we learn from you and other participants, we will plan a better way of providing abortion services in the future. Often, policies are made keeping in the broader population in mind but it is essential how they are implemented at local level where your role is most important. So, today, we are here to learn about ‘your’ experiences and thoughts and abortion care.

In order to make this discussion more meaningful and focused, we will have some guidelines that we would like to read before we start the discussion**-**

- *Your Participation is voluntary in this discussion.*
- *There are no right or wrong answers. For us, everyone’s views are important.*
- *One person should speak at a time.*
- *Everything you will tell or discuss in this discussion will be kept confidential and only be used for research purposes. So, we expect you to speak without any hesitation.*
- *What you say will be part of collective view, and your identity will be kept confidential.*
- *Please feel free to agree or disagree with each other respectfully*.

With this note, let us start the discussion-

A. **Background and role**
1. Can you introduce yourself one by one?

*[****Probe:*** *Name, village]*

2. How long have you been working in your role as a community health worker?

*[****Probe:*** *Month; Year]*

B. **Knowledge and awareness**
3. Can you describe different ways women in your area seek abortion services? What role, if any, do you play in this process?
4. What do you know about abortion? What are the local terms used for it?

*[****Probes:*** *Definitions of Abortion; Understanding in terms of gestational age; when do a women choose to seek abortion? Prevalent Reasons for abortion in the village or community?]*

5. Have you received any Information, Education and Communication (IEC) material for increasing awareness about abortion and contraceptive methods?

**[*Probes:*** *Poster, Leaflet or any other reference material]*

6. Are there any myths or misinformation in the community about abortion or abortion pills that you encounter?
7. Are you aware about any traditional methods/medicines that women use at local level to abort their pregnancy?

 [Probes: *home remedies, traditional healers, chemist shops*]

C. **Community Perception and Social Context**
8. How do women feel about seeking abortion, are there any concerns, fears, or social issues involved?
9. Whose opinion matters most in a woman’s decision to seek abortion (husband, in-laws, elders, religious leaders)? How do these views affect decisions?
10. How do families or partners generally respond when a woman decides to terminate a pregnancy?
11. Are there particular groups who face more challenges in accessing abortion?

*[****Probes:*** *Adolescents, unmarried women, victims of domestic or sexual violence]*

12. What are the major reasons that stop women from accessing safe abortion services in your village or community?

*[****Probes:*** *privacy, distance, cost, stigma, lack of information, health system issues]*

13. Are there groups of women who face more difficulties in accessing abortion? [adolescents, unmarried, migrants, lower caste/tribal, and women with disabilities]
D. **Practices and care pathways**
14. What do you do when a woman comes to you seeking abortion service?

*[****Probes:*** *Confidentially provide information, Do urine pregnancy test, Provide Medicine, Counsel, Refer or suggest to visit hospital, Take her to hospital]*

15. Are you involved in providing post-abortion care services? If yes, what post-abortion care services do you provide to the patients?

*[****Probe:*** *post abortion follow up and counselling, contraceptives]*

16. How do you know who need post-abortion care?

*[****Probe:*** *Are you informed by CHO, MPHW (F), Pharmacists, about patient who need post-abortion care?)*

17. When women in your area want to terminate a pregnancy, where do they usually go first?

*[****Probes:*** *Family, ASHA, AWW, Public Health Facility, Private clinics or facility, do they prefer management at home or at facility]*

18. How do you provide information or referrals related to abortion care?

*[****Probes:*** *Through IEC Material, Counselling, and Detail about doctor or hospital]*

19. Have you ever had to adjust or change your approach when supporting a woman seeking abortion? Can you describe?
E. **Training, Challenges, and Support Systems**
20. How many of you have received or attended any training or workshop to assist women seeking abortion services? Can you talk a little bit about when and where was it held and what were the major components of that training?

*[****Probes:*** *Training Centre, No. of Training days, given Travel Allowance (TA)/Dearness Allowance (DA), was the training useful? Component: training for confidential counselling; Promoting post abortion care and contraception]*

21. What are the challenges you face in promoting safe, evidence-based abortion care in your work?

*[****Probes:*** *training, guidelines, support from medical staff, resistance from families]*

22. What has helped you in guiding women to safe abortion care?
23. Are there any support systems

*[****Probe:*** *like doctors, supervisors, nobutn-government organizations (NGOs) you can rely on when you need help with abortion-related questions?]*

F. **Future Needs and Improvements**
24. What would help you support women better when it comes to abortion care?
25. What kinds of information, tools, or trainings do you think should be provided to health workers like yourself?
26. What one change would make it easier for women to access safe abortion care in your community/facility?”

**Closing Remark**

Thank you all for sharing your thoughts and experiences. Your insights are very valuable and will help improve services and support for the community.”
