# Supplementary material for: Status, barriers and facilitators of comprehensive abortion care services in rural India: A multicenter mixed-methods study protocol
Source: PLoS One. 2026 Sep 16;21(9):e0358274. doi: 10.1371/journal.pone.0358274 (PMC13581207; doi:10.1371/journal.pone.0358274)
Supplement: Supplementary file 5 [file pone.0358274.s002.zip › CAC IDI_guide for Women.docx]

**IDI Guide for Women**

**Study Title: Status, Barriers and Facilitators of Comprehensive Abortion Care Services: An MRHRU Based Study**

**Objective:** To understand women’s experiences with seeking CAC services: how decision around abortion care was made, what kind of care was received, where it was accessed and what factors influenced the experience of seeking CAC services.

**Introduction**

Thank you for taking the time to speak with me today. My name is [interviewer name], and I am part of the ICMR team. Before we begin, can I please confirm that you have received a copy of the study information sheet and consent form?

As a reminder, this project aims to understand the circumstances, decisions, care-seeking behaviours, and experiences related to abortion. We are conducting this study to identify the factors that influence how people access abortion-related care, the quality of care received, any challenges faced, and how the health care provision can be improved to better support individuals in similar situations. Our goal is to learn from your experience to strengthen the healthcare system and ensure people have access to safe, respectful, and appropriate care.

We want to understand things from your perspective and every response is valuable for this study. Your responses will be kept confidential and only be used for research purposes. We also assure you that your identity will not be linked to any of the findings. The interview will take approximately 45 minutes, depending on how much you'd like to share. Before we begin, I would like to confirm - are you comfortable with this space, and do you feel it is private enough for you to speak openly for the next 30–45 minutes? I would also like to request your permission to audio record our conversation. This will help me capture your responses accurately and focus on listening to you rather than taking detailed notes. Is it okay for me to start recording now?

Thank you.

I**nformation to collect (before starting):**

**Informant Details**

| Name |  |
| --- | --- |
| Age |  |
| Education |  |
| Village & Block |  |
| Date of Interview |  |
| Time since last Medical termination of pregnancy (MTP)/abortion |  |

**A. Basic Introduction [Previous History and possible reason for MTP/Abortion]**

1. Can you tell me about yourself?

[Probe: Age, marital status, age of marriage, family type, age at first conception,]

2. Please tell us about your pregnancies?

[Probe: How many times have you conceived? Status of previous children/ the gap period between the marriage and first conception and all the subsequent conceptions along with the outcome status (gestational age, live birth, intrauterine deaths, Neonatal deaths etc.)]

3. When and how did you come to know about the last pregnancy that was terminated?

[Probe: when: day, week, or month since last LMP, how: after missing periods, after experiencing dizziness, vomiting , fainting & weakness, BP, constant pain, feeling nausea, method of pregnancy test: urine examination using pregnancy kit, after visiting doctor, after ultrasound]

4. How did you feel after you came to know of this pregnancy?

[Probe: Expected, Unexpected, Happy, Shocked, wanted to have a baby, did not want to have a baby, were not ready to have a child]

5. When and whom did you first talk about this pregnancy? And what was the initial reaction?

[Probe: Husband, mother, friend, sister in law, no one]

**B. Reasons for terminating pregnancy**

6. Can you tell me about how the decision around this pregnancy was made?”

[Probe: unplanned, not ready, already had the desired number of children, Last baby too young and still breastfeeding, facing any health issue, on medication, Fear of congenital deformation or any other fear, unwillingness of husband or family members]

7. Did you make this decision on your own, or did someone else help you with it?

[Probe: Husband or Partner, In-laws, other family members, ASHA worker, RMP, Doctor, religious leaders]

**C. Knowledge of service & methods**

8. Did you previously know of any way to terminate this pregnancy?

[Probe: traditional methods, medical methods using medicine, surgical methods]

9. Who was your source of information?

[Probe: through ASHA, internet, friends, family member, previous experience]

**D. Care seeking behaviour**

10. What method did you choose for your abortion?

[Probe: What did you do to abort your pregnancy? Did you terminate by yourself at home? Had you taken any medicines or something else? What did you take? Where did you get it/Who brought it for you? Did you go somewhere? Did anyone accompany you for care if you went to a facility? Were some kind of instruments used? If it was a spontaneous abortion, how was the process completed – completed spontaneously or by medications or by surgical methods?]

11. If you went to a facility, was it a public facility or a private facility? What was the reason behind choosing the facility?

[Probe: Distance, privacy and confidentiality, cost, service provider’s gender, any other reason]

12. What was doctor’s response when you wished to abort your pregnancy at the facility?

[Probe: Asked reason, talked about the different procedures or methods, required time and risk, asked for a guardian to be present]

**E. Choice of abortion method**

13. Can you describe how did the doctor proceed with the procedure?

[Probe: after consultation with you, your husband or family member, after taking medical history, after physical examination, after getting some test done, after getting some forms signed]

14. Were you informed about different abortion methods (medicines or procedure), time required and risks involved before the doctor aborted your pregnancy?
15. Who suggested the method that was finally used?

[Probe: Someone in the house, a medical practitioner, knew it by yourself]

16. Were you given options about how the abortion would be done? If you think back now, would you prefer the same method again or a different method and why?

[Yes, No. If yes, why and if no, why?]

**F. Procedure and Post abortion experience**

17. Can you describe what happened during the abortion, what was done and how you felt?

[Probe: Privacy, respect, dignity, consent, Pain relief, hygiene, counselling, post-abortion contraception offers or refusal, Complications, follow-up, emergency care if needed]

18. Did you receive any care or advice after the procedure?

[Probe: did the doctor say anything? Did he give anything? What did he give? Did he tell you to follow some precautions? Was any documentation required? - ID proof, own or partner’s consent, signature]

19. Please tell us about any post-abortion care service that was provided to you.

[Probe: Follow up check-up, counselling services, family planning or contraceptive services, the services were provided at facility or at home by ASHA?]

**G. Perceived barriers**

20. What was the most difficult/challenging part of the whole experience?

[Probe: deciding to continue or abort, identifying a method and facility, long and painful procedure, cost, stigma, distance]

21. What helped you the most?

[Probe: Husband and family support, ASHA or care provider’s support, medical counselling, service availability at the facility,]

22. Some women use traditional medicines or pills from chemists. From your perspective, what makes women choose these options instead of going to a facility?”
23. In your opinion, what should be done to improve the abortion care facility in your village/community?

[Probe: Attitude of providers, Cleanliness, decrease in wait time, gender of provider, Suggestions on information, support, accessibility, distance from facility, decrease in cost]

24. If you were to face this situation again, what would you like to be different in how services are provided?”
25. Is there anything else you would like to share from your experience or someone else you know who went through such experience?
26. What one change would make it easier for women to access safe abortion care in your community/facility?”

**Closing Remarks**

Thank you very much for your time and for sharing your experiences. Your input is very important and will help improve reproductive health services for women in your community.
