# Supplementary material for: Status, barriers and facilitators of comprehensive abortion care services in rural India: A multicenter mixed-methods study protocol
Source: PLoS One. 2026 Sep 16;21(9):e0358274. doi: 10.1371/journal.pone.0358274 (PMC13581207; doi:10.1371/journal.pone.0358274)
Supplement: Supplementary file 5 [file pone.0358274.s002.zip › CAC IDI_Guide for Facilty Incharge or Manager.docx]

**In-depth Interview Guide for Facility In-charge /Facility Manager**

**Study Title:** Status, Barriers, and Facilitators of Comprehensive Abortion Care Services: An Model Rural Health Research Units (MRHRU) Based Study

**Objective:** **To understand the overall functioning of the facility** with respect to the provision of Comprehensive Abortion Care (CAC) services, including availability of essential infrastructure, staffing, workload, and coordination.

**Introduction**

Thank you for taking the time to speak with me today. My name is [interviewer name], and I am part of the Indian Council of Medical Research (ICMR) team. Before we begin, can I please confirm that you have received a copy of the study information sheet and consent form?

As a reminder, this project aims to understand the circumstances, decisions, care-seeking behaviours, and experiences related to abortion. We are conducting this study to identify the factors that influence how people access abortion-related care, the quality of care received, any challenges faced, and how the health care provision can be improved to better support individuals in similar situations. Our goal is to learn from your experience to strengthen the healthcare system and ensure people have access to safe, respectful, and appropriate care.

We want to understand things from your perspective and every response is valuable for this study. Everything you share with us will be treated with full discretion. Your responses will be kept confidential and only be used for research purposes. We also assure you that your identity will not be linked to any of the findings. The interview will take approximately 45 minutes, depending on how much you would like to share. May I check if you are free to speak for that amount of time now?

I would also like to request your permission to audio record our conversation. This will help me capture your responses accurately and focus on listening to you rather than taking detailed notes. Is it okay for me to start recording now?

Thank you.

I**nformation to collect (before starting):**

| **Name** |  |
| --- | --- |
| **Age (in years)** |  |
| **Education Qualification** |  |
| **Year of Experience** |  |
| **Facility Name/Level** |  |
| **Facility Area (Block )** |  |
| **Date of the interview** |  |

A. **Facility Overview**
1. Could you please briefly tell us about your facility type and your role here?

*[Probe: PHC/CHC/SDH/DH; manager, in-charge,]*

B. **Abortion Services**
2. Can you tell if the abortion or CAC services currently provided at your facility?

*[Probe: For which gestational period (up to 9 weeks, up to 12 weeks, up to 24 weeks; Beyond 24 weeks)? If not, where are women referred?]*

C. **Essential Requirements**
3. Can you tell what the essential requirements for providing CAC services are? Are they available at your facility?

*[Probe: Equipment, medicines, trained staff, which items are missing, and how do you manage in emergencies?]*

4. Does this facility have a gynaecological examination or labour table for pregnancy termination procedures?

[Probes: Is it in functional condition?]

5. Do you have an ultrasound facility within your premises?

*[Probe: If not, where are women sent and how is this managed?]*

6. Is there a blood storage unit or blood bank at this facility?

*[Probe: How do you arrange blood in emergencies? Any challenges?]*

D. **Emergency Preparedness**
7. What resuscitation equipment and sterilization facilities are available here?

[Probes: Are oxygen and ambu-bags available and functional? Do you have sterilization instruments like autoclave, boiler available here?]

E. **Counselling Space**
8. Do you have a separate room or private space for counselling?

*[Probe: How do you ensure privacy and confidentiality if space is limited?]*

F. **Infrastructure Reliability**
9. How reliable is electricity and water supply in your facility?

*[Probe: Are there backup systems (generator, inverter)? Do toilets have running water?]*

G. **IEC Material**
10. Do you have any CAC related standard IEC materials?

*[Probe: Site signage, posters on CAC services, posters on consequences of unsafe abortion procedures, posters related to post-abortion contraception, or information leaflets for giving to health workers at your facility?]*

11. If not, which materials related to CAC services are currently displayed in your facility? Are they visible and accessible to patients?
H. **Supply Chain**
12. Is the supply of essential CAC drugs and consumables (Mifepristone, Misoprostol, antibiotics, analgesics, gloves, and IV fluids)?

*[Probe: What do you do if there are shortages?]*

I. **Ambulance & Referral Linkages**
13. How do you manage referrals for abortion cases requiring higher-level care?

*[Probe: Ambulance availability, timeliness, cooperation with referral centres.]*

J. **Support & Coordination**
14. How responsive are district/state health authorities when you request additional support (equipment, supplies, training)? Do you get enough support from the authorities?

*[Probe: Example of when support was timely vs. delayed.]*

K. **Key Gaps & Improvements**
15. In your view, what are the two biggest gaps in CAC infrastructure here?

[Probe: lack of essential equipment, lack of trained staff, irregular supply of medicine, lack of support from high authorities, lack of essential fund]

16. From your perspective, what one change would make it easier for women to access safe abortion care at this facility?

**Closing Remark**

Thank you very much for your time and for sharing your experiences. Your input is very important and will help improve reproductive health services for women in your community.
