# Supplementary material for: Status, barriers and facilitators of comprehensive abortion care services in rural India: A multicenter mixed-methods study protocol
Source: PLoS One. 2026 Sep 16;21(9):e0358274. doi: 10.1371/journal.pone.0358274 (PMC13581207; doi:10.1371/journal.pone.0358274)
Supplement: Supplementary file 5 [file pone.0358274.s002.zip › CAC_KII_Guide for ANM & CHO.docx]

**Key Informant Interview Guide for Health Care Professionals**

**[Auxiliary Nursing Midwife (ANM) AND Chief Health Officer (CHO)]**

**Study Title: Status, Barriers, and Facilitators of Comprehensive Abortion Care Services: An Model Rural Health Research Units (MRHRU) Based Study**

**Objective:** To examine the role of ANMs and CHOs at Sub-Centres and Health and Wellness Centres in supporting Comprehensive Abortion Care (CAC), with a focus on pre- and post-abortion care, counselling, stigma navigation, and the societal and institutional challenges they encounter.

**Introduction**

Thank you for taking the time to speak with me today. My name is [interviewer name], and I am part of the Indian Council of Medical Research (ICMR) team. Before we begin, can I please confirm that you have received a copy of the study information sheet and consent form?

As a reminder, this project aims to understand the circumstances, decisions, care-seeking behaviours, and experiences related to abortion. We are conducting this study to identify the factors that influence how people access abortion-related care, the quality of care received, any challenges faced, and how the health care provision can be improved to better support individuals in similar situations. Our goal is to learn from your experience to strengthen the healthcare system and ensure people have access to safe, respectful, and appropriate care.

We want to understand things from your perspective and every response is valuable for this study. Everything you share with us will be treated with full discretion. Your responses will be kept confidential and only be used for research purposes. We also assure you that your identity will not be linked to any of the findings. The interview will take approximately 45 minutes, depending on how much you would like to share. May I check if you are free to speak for that amount of time now?

I would also like to request your permission to audio record our conversation. This will help me capture your responses accurately and focus on listening to you rather than taking detailed notes. Is it okay for me to start recording now?

Thank you.

I**nformation to collect (before starting):**

**Informant Details**

| **Name** |  |
| --- | --- |
| **Age (in years)** |  |
| **Education Qualification** |  |
| **Year of Experience** |  |
| **Facility Name/Level** |  |
| **Facility Area(village & Block )** |  |
| **Date of the interview** |  |

A. **Warm Up Questions**
1. What are the services currently being provided at this facility?
2. Can you tell me about your role at this facility?

*[Probes: Major responsibilities in general, Reproductive health services, Abortion- related services]*

3. How long have you been working at this facility and in this role?

*[Probes: Have you received any specific training related to abortion care or post-abortion care (e.g., CAC, Family Planning, counselling)]*

B. **Abortion Services**
4. Can you describe how do you support women seeking abortion services at your facility?

*[Probe: Provide information about the legal rights or options, Provide information about different methods of abortion service; provide counselling for unplanned pregnancy; counselling for Post abortion care, provide post-abortion emotional/psychosocial support]*

5. Do you provide any service related to comprehensive abortion care at this facility?

*[Probes: Yes; No; is this facility certified/designated for providing CAC services]*

6. Can you describe what services related to abortion are available at your facility, if any?”?

*[Probes: Pre -abortion Counselling, Medical method of abortion; Post-abortion follow up and Counselling; family planning services including counselling for use of contraceptives (condoms, pills, injectable, intrauterine contraceptive device (IUCDs, Sub-dermal implants), sterilisation)]*

7. What are the common circumstances in which pregnant women seek CAC service in your catchment are?

*[Probes: Unwanted pregnancy, Contraceptive failure, Genetic abnormality/foetal anomaly, Septic abortion, incomplete abortion; if other, please specify]*

8. Do you keep any record of women who visit your facility seeking CAC services? If yes, how?

*[Probes: Form II, Form III and Form C (Documentation and consent)]*

C. **Collaboration and Referrals (CHO & ANM Specific)**
9. What do you do after counselling a women for CAC services?

[*Refer to PHC, CHC and other higher facility*]

10. What process do you follow when referring the women to a CHC or a PHC?

*[Probes: Providing the reason why being referred to? Name of the facility where being referred, a referral letter? Informing the facility where referring, instruction for follow-ups]*

11. What are the major challenges you encounter when referring a person to another facility?

*[Probes: Unwillingness of the person or their family, fear of legal repercussion, gendered moral judgement, community surveillance & moral judgment, long distance, Poor coordination]*

12. How do you coordinate with the PHC medical officer, ASHA, or others in your facility for abortion-related cases?”
D. **Post-Abortion Care and Follow-Up**
13. Do you follow up with women who were referred or received abortion services, and if yes, in what ways?

[*Call them to revisit you after abortion; Contact them through phone; Visit them at home]*

14. What care is given to women after an abortion?

*[Probes: Counselling & reassurance; Checking Recovery; Contraception advice; Emotional Support]*

15. Are there any family planning services offered after an abortion? Can you specify?

*[Probes: Contraceptive methods (e.g., pills- Chhaya, condoms, intrauterine contraceptive device (IUCD), injectable- Antara), sterilisation*]

16. Do you have a separate space to provide counselling at your facility?

*[Probe: how do you maintain the privacy and confidentiality of the patient?]*

17. What is the average number of abortion cases that come to your facility in a day or a week?
18. Have you received any training for counselling the women seeking CAC services?

*[Probes: Attended a CAC meeting or workshop; received any counselling module or guideline?]*

19. Do you feel confident in counselling and supporting women seeking abortion services?

[Probes: what gives confidence, what makes it difficult, what additional support you need?]

20. What challenges do you face in providing abortion-related care?

*[Probes: Clinical, emotional, supply-related, manpower, legal, or cultural? Have you faced any challenge or criticism from the family or local community while providing counselling or care to a woman?]*

E. **Barriers and Community Response**
21. What are the perception and attitude of pregnant women of your area, towards comprehensive abortion care services?
22. Do you face resistance or stigma from the community or staff around abortion services? *[How do you deal with such situations?]*
23. Can you tell us the factors that prevent women from seeking abortion care at appropriate health facilities like CHC and PHC?
    *[Consulting Non-Registered Medical Practitioner, Distance, stigma, fear, lack of awareness,]*
24. How do you manage abortion cases that are exceptional in their nature?

[Probe*: Contact senior medical officers, refer to higher facility]*

25. Are you aware of any other prevalent traditional methods of abortion in the local community?

*[Probe: Traditional Practitioner i.e. Midwives, Hakim, Quacks, Over-the-counter pills from pharmacies etc.]*

26. What kind of support do you think women need most when coming for abortion services?

*[Probes: Personal Support, support from family, Emotional and Social Support, Communication/travel; is there anything that stops women from coming to the facility for abortion care?]*

27. What changes or improvements would help you provide better abortion care?

*[Probes: Training, More Training, supplies, staff support, infrastructure, awareness in community?]*

**Closing Remarks**

Thank you very much for your time and for sharing your experiences. Your input is very important and will help improve reproductive health services for women in your community.
