# Supplementary material for: Status, barriers and facilitators of comprehensive abortion care services in rural India: A multicenter mixed-methods study protocol
Source: PLoS One. 2026 Sep 16;21(9):e0358274. doi: 10.1371/journal.pone.0358274 (PMC13581207; doi:10.1371/journal.pone.0358274)
Supplement: Supplementary file 5 [file pone.0358274.s002.zip › CAC_KII_Guide for Health care Professionals.docx]

**KII Guide for Health Care Professionals**

**(Senior Medical Officer (SMO), Medical officer (MO), Gynaecologist/Obstetrician & Staff Nurse)**

**Study Title: Status, Barriers, and Facilitators of Comprehensive Abortion Care Services: An Model Rural Health Research Units (MRHRU) Based Study**

**Objective:** To understand how health care professionals (Senior medical officer, medical officers, Gynaecologist/Obstetrician & Staff Nurse appointed at public medical facilities provide or support Comprehensive Abortion Care (CAC) services, focusing on pre and post-abortion care, counselling, stigma navigation, and institutional challenges.

**Introduction**

Thank you for taking the time to speak with me today. My name is [interviewer name], and I am part of the Indian Council of Medical Research (ICMR) team. Before we begin, can I please confirm that you have received a copy of the study information sheet and consent form?

As a reminder, this project aims to understand the circumstances, decisions, care-seeking behaviours, and experiences related to abortion. We are conducting this study to identify the factors that influence how people access abortion-related care, the quality of care received, any challenges faced, and how the health care provision can be improved to better support individuals in similar situations. Our goal is to learn from your experience to strengthen the healthcare system and ensure people have access to safe, respectful, and appropriate care.

We want to understand things from your perspective and every response is valuable for this study. Everything you share with us will be treated with full discretion. Your responses will be kept confidential and only be used for research purposes. We also assure you that your identity will not be linked to any of the findings. The interview will take approximately 45 minutes, depending on how much you would like to share. May I check if you are free to speak for that amount of time now? Also, can you confirm that this is a private space where you feel comfortable speaking freely?

I would also like to request your permission to audio record our conversation. This will help me capture your responses accurately and focus on listening to you rather than taking detailed notes. Is it okay for me to start recording now?

Thank you.

I**nformation to collect (before starting):**

**Informant Details**

| **Name** |  |
| --- | --- |
| **Age (in years)** |  |
| **Education Qualification** |  |
| **Year of Experience** |  |
| **Facility Name/Level** |  |
| **Facility Area(village & Block )** |  |
| **Date of the interview** |  |

A. **Warm Up Questions**
1. What are the services currently being provided at this facility?
2. Can you tell me about your role at this facility?

[Probes: Major responsibilities in general, Reproductive health services, Abortion- related services]

3. How long have you been working at this facility and in this role?

[Probes: Have you received any specific training related to abortion care or post-abortion care (e.g., CAC, Family Planning, counselling)]

B. **Abortion Services**
4. Are abortion care services available at this facility?

[Probes: Yes; No; is this facility certified/designated for providing CAC services]

5. What kind of abortion services are provided at this facility?

[Probes: Medical abortion services (Mifepristone + Misoprostol); Surgical abortion services (Manual Vacuum Aspiration / Electric; Management of incomplete abortion (spontaneous or induced); Management of complications of abortion, Post-abortion follow up and Counselling; family planning services including sterilisation and counselling for use of contraceptives (condoms, pills, injectable, intrauterine contraceptive device (IUCDs, Sub-dermal implants), sterilisation)]

6. What are the common conditions for which pregnant women seek CAC service at your facility?

[Probes: Unwanted pregnancy, Contraceptive failure, Genetic abnormality/foetal anomaly, Septic abortion, incomplete abortion etc.]

7. Can you share about the procedures or assessments done before abortion?

[Probes: History taking; Physical examination; Pelvic examination; Laboratory investigations; Pregnancy testing; checking gestational age, Screening or assessing eligibility of the women for abortion (e.g., gestational age, signs of complications), Pre- Abortion counselling]

**8**. What kind of information is provided to women seeking abortion services?

[Probe: Availability of printed Information, Education and Communication (IEC) Material or Comprehensive Abortion Care (CAC )or world health organization (WHO) Guideline; Information about the legal rights or options, Counselling for unplanned pregnancy; Counselling for Post abortion or post miscarriage care, post-abortion emotional/psychosocial support, Counselling in private and confidential setting]

**9**. Is ultrasound or lab testing available here? Are there any mandatory tests and procedures when someone comes to seek abortion care? If yes, can you please specify the tests?

[Probes: Haemoglobin Test, Differential Count (DC), Total Leukocyte count (TLC), Urine Test for routine and microscopy, Random Blood Sugar (RBS), Blood group Test, Urine (for pregnancy test with Nischay kit), Hepatitis B (HBsAg), HIV test]

**10**. Who usually performs these procedures? What is your role in provision of abortion care in the facility?

[Probes: Role in administering medical abortion (Mifepristone and Misoprostol) & surgical procedures (e.g., performing/assisting during Manual Vacuum Aspiration or MVA), Administrative/management role in provision of abortion care in the facility]

**11**. How do you keep the record of abortions at your facility?

[Probes: Form II, Form III and Form C (Documentation and consent]

C. **Collaboration and Referrals (CHO & ANM Specific)**

**12.** In what cases do you refer the person to the other facilities? What process do you follow when referring to another facility?

[Probes: Providing the reason why being referred to? Name of the facility where being referred, a referral letter? Informing the facility where referring, instruction for follow-ups]

**13.** What are the major challenges you encounter when referring a person to another facility?

[Probes: Unwillingness of the person or their family, fear of legal repercussion, gendered moral judgement, community surveillance & moral judgment, long distance, Poor coordination]

D. **Post-Abortion Care and Follow-Up**

**14.** What care is given to women after an abortion?

[Probes: Medicines for pain relief, antibiotics; Observation; Counselling; Follow	up care]

**15.** Are there any family planning services offered after an abortion? Can you specify?

[Probes: Contraceptive methods (e.g., pills- Chhaya, condoms, intrauterine contraceptive device (IUCD), injectable- Antara), sterilisation]

**16.** Do you have a separate space to provide counselling at your facility?

[Probe: how do you maintain the privacy and confidentiality of the patient?]

**E. Supplies and Infrastructure**

**17.** What are the necessary supplies and medications required in abortion services? Are they available when needed at this facility?

[Probes: Gloves, mask, sterilized instruments, abortion pills, medications for pain relief, antibiotics. Have there been times when supplies were lacking? If yes, since when? How did you manage?]

**18**. Have there been times when supplies were not available? How did you manage in such situations?

[can you list the missing supplies, how often does it happen?]

**19.** What is the average number of abortion cases that come to your facility in a day or a week? Do you feel there is adequate staff to handle the workload for abortion-related services?

[Probes: Again, how are CAC cases documented? What kind of support or supervision do you receive?]

**20.** Have you received any training particularly for abortion care service?

[Probes: CAC Training; Medical Termination of Abortion (MTA) Training; Medical Method of Abortion (MMA) Training; Infection prevention procedures during abortion care, what steps do you take to ensure cleanliness and safety during care?]

**21**. Who is involved in providing abortion and post-abortion care at this facility?

[Probes: Doctors, Nurses (Nursing Officers), CHO, MPHW (F) Accredited Social Health Activist (ASHAs), counsellors]

**22**. What challenges do you face in providing abortion-related care?

[Probes: Clinical, emotional, supply-related, manpower, legal, or cultural? Have you faced any challenge or criticism from the family or local community while providing counselling or care to a woman?]

F. **Barriers and Community Response**

**23**. What are the perception and attitudes of the family or the community toward comprehensive abortion care services in your catchment area?

**[**Awareness of legal abortion rights; Misconceptions or myths (safety, morality, legality) Stigma or judgment toward women seeking care; Family/spousal influence on decision-making; Preference for private vs. public facilities; Reliance on traditional/unregistered providers; Differences by age, caste, religion, or education]

**24.** Do you face resistance or stigma from the community or staff around abortion services? [Probe: How do you deal with such situations? Can you recall any such instance for us?]

25. How supportive is your facility leadership in prioritizing abortion services compared to other services?

26. How do colleagues and staff at your facility generally feel about abortion services? How does this affect your work?

**27**. Can you tell us the factors that prevent women from seeking abortion care at public health facility?
[Consulting Non-Registered Medical Practitioner, Distance, stigma, fear, lack of awareness,]

**28**. How do you manage abortion cases that are exceptional in their nature?

[ Probe i.e. Cases of domestic violence ; Cases of teenage/minor pregnancy ; Cases of congenital diseases or foetal abnormalities ; Case of rape victim ; Cases with high risk pregnancy ; Cases where respondent does not wish to inform family members]

**29.** Are you aware of any other prevalent traditional methods of abortion in the local community?

[Probe: Traditional Practitioner i.e. Midwives, Hakim, Quacks, Over-the-counter pills from pharmacies etc.]

**30.** What kind of support do you think women need most when coming for abortion services?

[Probes: Personal Support, support from family, Emotional and Social Support, Communication/travel; is there anything that stops women from coming to the facility for abortion care?]

**31.** What changes or improvements would help you provide better abortion care?

[Probes: Training, More Training, supplies, staff support, infrastructure, awareness in community?]

**Closing Remarks**

Thank you very much for your time and for sharing your experiences. Your input is very important and will help improve reproductive health services for women in your community.
