# Supplementary material for: Status, barriers and facilitators of comprehensive abortion care services in rural India: A multicenter mixed-methods study protocol
Source: PLoS One. 2026 Sep 16;21(9):e0358274. doi: 10.1371/journal.pone.0358274 (PMC13581207; doi:10.1371/journal.pone.0358274)
Supplement: Supplementary file 5 [file pone.0358274.s002.zip › CAC facility tool - Sub-centre.pdf]

SUB - CENTRE				
**No.**	**Question**	**Answer**		**Skip**
**1. FACILITY IDENTIFIERS**				
1.1	Type of Facility			
1.2	Name of the region			
1.3	Name of the district			
1.4	Name of the respondent			
1.5	Designation of the respondent			
1.6	Date of interview (DD/MM/YYYY)			
**2. INFORMATION AND COUNSELLING**				
2.1	Does this facility provide any abortion-related information and counselling to abortion care clients?	YES___________1<br>NO____________2		
**3. SERVICES PROVIDED**				
3.1	Does this facility offer post-abortion contraceptive services such as providing condoms, oral contraceptive pills, injectables, IUCDs etc.?	YES___________1<br>NO____________2		
**4. HUMAN RESOURCE**				
4.1	Are the CHOs and ASHAs trained for CAC?			
		Staff available, document seen	Staff available, document not seen	Staff not available
4.1.1	CAC trained CHO	1	2	3
4.1.2	CAC trained ASHAs	1	2	3
**5. DOCUMENTS / REFERRALS**				
5.1	Please show me the following forms, documents and registers related to abortion and post-abortion services that are maintained in this facility.			
		Yes, Observed	Yes reported, not seen	Not available
5.1.1	Referral register	1	2	3
