# Supplementary material for: Status, barriers and facilitators of comprehensive abortion care services in rural India: A multicenter mixed-methods study protocol
Source: PLoS One. 2026 Sep 16;21(9):e0358274. doi: 10.1371/journal.pone.0358274 (PMC13581207; doi:10.1371/journal.pone.0358274)
Supplement: Supplementary file 5 [file pone.0358274.s002.zip › CAC facility tool - PHC.pdf]

PHC			
**No.**	**Question**	**Answer**	
**1. FACILITY IDENTIFIERS**			
1.1	Type of Facility		
1.2	Name of the region		
1.3	Name of the district		
1.4	Name of the respondent		
1.5	Designation of the respondent		
1.6	Date of interview (DD/MM/YYYY)		
**2. INFORMATION AND COUNSELLING**			
2.1	Does this facility provide any abortion-related information and counselling to abortion care clients?	YES____________1<br>NO_____________2	

**3. SERVICES PROVIDED**			
		YES	NO
3.1	Does this facility offer medical abortion services for ≤12 weeks of gestation?	1	2
3.2	Does this facility offer surgical abortion services for ≤12 weeks of gestation?	1	2
3.3	Does this facility offer services for the management of incomplete abortion (both spontaneous and induced)?	1	2
3.4	Are pregnancy test kits available to diagnose pregnancy	1	2
3.5	Is ultrasonography available in the facility to diagnose pregnancy/incomplete abortion etc.?	1	2
3.6	Does the facility offer post-abortion follow up services?	1	2
3.7	Does the facility offer post-abortion contraceptive services such as providing condoms, oral contraceptive pills, injectables, IUCDs, sterilisation services etc.	1	2

**4. READINESS**				
	Request to check the drug registers/documents in the storage facility.<br>"Now I would request you to show me the drug register or related document in the storage facility."			
		(A) AVAILABLE		
**4.A**	**Essential drugs/consumables**	Observed	Reported, Not seen	Not available
4.A.1	Antibiotics	1	2	3
4.A.2	Analgesics	1	2	3
4.A.3	Tab Misoprostol	1	2	3
4.A.4	Tab Mifepristone	1	2	3
4.A.5	Injection Oxytocin	1	2	3
4.A.6	I/V fluids	1	2	3
**4.B**	**Emergency drugs**	Observed	Reported, Not seen	Not available
4.B.1	Inj. Diazepam	1	2	3
4.B.2	Inj. Adrenaline	1	2	3
4.B.3	Inj. Aminophylline	1	2	3
4.B.4	Inj. Avil/Phenergan	1	2	3
4.B.5	Inj. Hydrocortisone	1	2	3
4.B.6	Inj. Dopamine	1	2	3

		(A) AVAILABLE			Skip	(B) FUNCTIONING		
4.C	**Equipment**	Observed	Reported, Not seen	Not available		Yes	No	Don't Know
4.C.1	MVA/EVA tray (gloves, sim's speculum, valsellum, sponge holding forceps, MVA aspirator, suction machine/foot pump, cannulae, dilators, curette, syringes, cotton/gauze etc.)	1---->B	2----->B	3	---->4.C.2	1	2	3

4.C.2	Bed	1---->B	2----->B	3	---->4.C.3	1	2	3
4.C.3	Sterlisation equipment	1---->B	2----->B	3	---->4.C.4	1	2	3
4.C.4	Resuscitation equipment and Anaesthetic equipment	1---->B	2----->B	3	---->5	1	2	3

5. INFRASTRUCTURE

	Does this facility have an abortion procedure room/operation theatre where abortion services, including incomplete abortion, are performed? May I see the room? Does the facility have the following?		
		(A) AVAILABLE	
		Yes	No
5.1	Adequate seating arrangement for women and the person accompanying	1	2
5.2	Door/Screens/Curtains for privacy	1	2
5.3	Display of IEC	1	2
5.4	Site Signage	1	2
5.5	Examination table with footstep	1	2
5.6	Functional toilets with water supply	1	2
5.7	Uniterrupted Electric Supply/Power backup	1	2

6. DOCUMENTS / REFERRALS

	Please show me the following forms, documents and registers related to abortion and post-abortion services that are maintained in this facility.					
		Yes Observed	Yes reported, not seen	Not available	Not Applicable (NA)	Skip
6.1	Form C	1	2	3	4	
6.2	Form I,E &D *	1	2	3	4	--------> 6.3
6.3	Form III - Admission register	1	2	3	4	
6.4	Monitoring tool for CAC services	1	2	3	4	
6.5	Referral register	1	2	3	4	
6.6	Form II	1	2	3	4	

7.HUMAN RESOURCE (TRAINED)

	Are there a CAC Trained MO and a CAC trained nursing staff?			
		Staff available, document seen	Staff available, document not seen	Staff not available
7.1	CAC trained MO	1	2	3
7.2	CAC trained Nursing staff	1	2	3
