# Supplementary material for: Status, barriers and facilitators of comprehensive abortion care services in rural India: A multicenter mixed-methods study protocol
Source: PLoS One. 2026 Sep 16;21(9):e0358274. doi: 10.1371/journal.pone.0358274 (PMC13581207; doi:10.1371/journal.pone.0358274)
Supplement: Peer review history

# Author comment: Author response to Decision Letter 0

**DOI:** 10.1371/journal.pone.0358274.r001

9 Apr 2026

Attachment Submitted filename: Response-Editorial Comment_3.0.docx

# Peer review: Decision Letter 0

**DOI:** 10.1371/journal.pone.0358274.r002

**Authors:** Arun Kumar Sharma

13 May 2026

Dear Dr. Mukerjee,

Please submit your revised manuscript by Jun 27 2026 11:59PM. If you will need more time than this to complete your revisions, please reply to this message or contact the journal office at plosone@plos.org. When you're ready to submit your revision, log on to https://www.editorialmanager.com/pone/ and select the 'Submissions Needing Revision' folder to locate your manuscript file.

- A letter that responds to each point raised by the academic editor and reviewer(s). You should upload this letter as a separate file labeled 'Response to Reviewers'.
- A marked-up copy of your manuscript that highlights changes made to the original version. You should upload this as a separate file labeled 'Revised Manuscript with Track Changes'.
- An unmarked version of your revised paper without tracked changes. You should upload this as a separate file labeled 'Manuscript'.

If you would like to make changes to your financial disclosure, please include your updated statement in your cover letter. Guidelines for resubmitting your figure files are available below the reviewer comments at the end of this letter.

If applicable, we recommend that you deposit your laboratory protocols in protocols.io to enhance the reproducibility of your results. Protocols.io assigns your protocol its own identifier (DOI) so that it can be cited independently in the future. For instructions see: https://journals.plos.org/plosone/s/submission-guidelines#loc-laboratory-protocols. Additionally, PLOS ONE offers an option for publishing peer-reviewed Lab Protocol articles, which describe protocols hosted on protocols.io. Read more information on sharing protocols at https://plos.org/protocols?utm_medium=editorial-email&utm_source=authorletters&utm_campaign=protocols.

As the corresponding author, your ORCID iD is verified in the submission system and will appear in the published article. PLOS supports the use of ORCID, and we encourage all coauthors to register for an ORCID iD and use it as well. Please encourage your coauthors to verify their ORCID iD within the submission system before final acceptance, as unverified ORCID iDs will not appear in the published article. *Only*  the individual author can complete the verification step; PLOS staff *cannot*  verify ORCID iDs on behalf of authors.

We look forward to receiving your revised manuscript.

Kind regards,

Arun Kumar Sharma

Academic Editor

PLOS One

Journal Requirements:

When submitting your revision, we need you to address these additional requirements.

1. Please ensure that your manuscript meets PLOS ONE's style requirements, including those for file naming. The PLOS ONE style templates can be found at

https://journals.plos.org/plosone/s/file?id=wjVg/PLOSOne_formatting_sample_main_body.pdf and

https://journals.plos.org/plosone/s/file?id=ba62/PLOSOne_formatting_sample_title_authors_affiliations.pdf

2. We note that the grant information you provided in the ‘Funding Information’ and ‘Financial Disclosure’ sections do not match.

When you resubmit, please ensure that you provide the correct grant numbers for the awards you received for your study in the ‘Funding Information’ section.

3. Thank you for stating the following financial disclosure:

“It is funded by the Indian Council of Medical Research (ICMR) Delhi (Grant No: 5/7/Intramural/Dr_Bijaya_Mishra). Representatives from ICMR contributed to the conceptualization and protocol development and will continue to be part of the study by providing oversight through supervision and monitoring. All data generated from the study will be stored electronically at ICMR, and the institute retains the rights to the data.   “

Please state what role the funders took in the study. If the funders had no role, please state: "The funders had no role in study design, data collection and analysis, decision to publish, or preparation of the manuscript."

If this statement is not correct you must amend it as needed.

Please include this amended Role of Funder statement in your cover letter; we will change the online submission form on your behalf.

4. Please include captions for your Supporting Information files at the end of your manuscript, and update any in-text citations to match accordingly. Please see our Supporting Information guidelines for more information: http://journals.plos.org/plosone/s/supporting-information.

5. If the reviewer comments include a recommendation to cite specific previously published works, please review and evaluate these publications to determine whether they are relevant and should be cited. There is no requirement to cite these works unless the editor has indicated otherwise.

**Additional Editor Comments:**

A very important research is being proposed by the authors. This has a potential to help us understand the status, barriers and facilitators of comprehensive abortion care services in rural areas. Being a multi centre study, the results will highlight the scenario of CAC services for the policy makers to improve the services. The authors may respond to following observations:

1.Title: It is a multi center study and not multi centric study.

2. Methods

A. Why unmarried women are excluded. The riask of unsafe abortion and barriers to CAC among unmarried women are equally important.

B. Legally defined age is 18 to 49 but pregnancy below 18 years is not illegal per se. By excluding the 15-18 age group, in Indian conext, a key and vulnerable beneficiary is getting excluded from the study.

C. Sample size: Assuming 50% prevalence across all selected MRHRUs is an untenable value for sample size calculation. In absence of relevant literature, pilot study is undertaken to calculate sample size. If the results show very low prevalnce say < 10% or very high prevalence, say >90%, the sample size estimation will become null and void.

D. What is the rationale for design effect of 2?

F. In a cross sectional survey , there is no utility of accounting for attrition.

G. For qualitative component, age range is described as 15 to 49, contradictng the legal age of 18 to 49 years

H. How can multivariable rgression models be generated if there are no a priori hypotheses stated and sample sizes are calculated as per hypotheses?

I. How missing data will be handled, should be explained.

[Note: HTML markup is below. Please do not edit.]

Reviewers' comments:

Reviewer's Responses to Questions

**Comments to the Author**

1. Does the manuscript provide a valid rationale for the proposed study, with clearly identified and justified research questions?

Reviewer #1: Yes

Reviewer #2: Yes

**********

2. Is the protocol technically sound and planned in a manner that will lead to a meaningful outcome and allow testing the stated hypotheses?

Reviewer #1: Yes

Reviewer #2: Yes

**********

3. Is the methodology feasible and described in sufficient detail to allow the work to be replicable?

Reviewer #1: Yes

Reviewer #2: Yes

**********

4. Have the authors described where all data underlying the findings will be made available when the study is complete?

The PLOS Data policy

Reviewer #1: Yes

Reviewer #2: Yes

**********

5. Is the manuscript presented in an intelligible fashion and written in standard English?

Reviewer #1: Yes

Reviewer #2: Yes

**********

Please use the space provided to explain your answers to the questions above and, if applicable, provide comments about issues authors must address before this protocol can be accepted for publication. You may also include additional comments for the author, including concerns about research or publication ethics.

You may also provide optional suggestions and comments to authors that they might find helpful in planning their study.

Reviewer #1: Thank you for your submission. This is a well written protocol for a well planned multi-centric study, which when done well, will provide valuable insights for implementation of the model 0, as stated. Please address the following issues, to make the manuscript a comprehensive read for the audience, so that it may stand alone on its own -

1. Elaborate more on the selection and deployment of data collectors/ interviewers/ moderators for qualitative data collection - what kind of relationship do they share with the community/ what training they have undergone/ what stakeholders they will be engaging with to make them suitable to undertake such sensitive conversations in their respective areas? This may also include their relevant past experience in the same area.

2. How are the live-in relationships in Maharashtra area being defined? Is there a time-based criterion for inclusion, or any criteria? The exception has only been mentioned, not explained.

3. How were the questions for moderator/ facilitator guide(s) for KII and FGDs arrived at? Was the CFIR framework used for the design of the guide(s) as well? What were the key literature findings that guided the choice of questions.

4. It is a good practice in qual research for moderator/ facilitators to reconvene after FGDs to tweak the guide(s) based on their subsequent experiences of FGDs. Some new areas may come up during FGD, which make good addition to the guide for subsequent discussions. While the authors mention maintaining an audit trail throughout the study, it is not clear of the same extends to this specific aspect of refining the tool.

Reviewer #2: Manuscript number: PONE-D-26-10674

Reviewer’s Comments:

The Comprehensive Abortion Care (CAC) tool from India's National Health Mission is a vital guide for ensuring safe abortion through improved planning and health system requirements. As the authors note, only a few studies demonstrate that expanding facilities is closely linked to increased availability of abortion care services.

Given that facility readiness is a critical aspect of successful implementation of CAC service in India and that implementation research plays an important role in adopting evidence-based services in India, this study—utilizing model rural health research units—is highly relevant. We consider this study protocol for publication with some minor revisions to enhance its clarity and contribution.

Introduction section

The manuscript provides a detailed rationale for generating evidence regarding facility readiness and care-seeking experiences in rural settings. To further strengthen this section:

• The authors should highlight the necessity of conducting this mixed-method study as a multi-centre initiative.

• To assist an international audience, elaborating on the specific contextual similarities and differences across the various study sites in India will support the rationale.

Methods section

• The data collection methods are well-documented, and the involvement of multiple stakeholders is a significant strength. Data collection methods has been well explained and documented. Involving multiple stakeholders is an important step. The inclusion of population differences for the Maharashtra study site in the eligibility criteria is noted and appreciated.

However, the following points regarding big qualitative data and sampling should be addressed:

• Predicting in advance, how much data will be enough is a requirement for a-priori sample size calculation and funding requirements. While it is mentioned that sampling will be guided by thematic saturation, the authors must be aware and mention somewhere in the section on “Qualitative sampling” that recruitment methods will facilitate varied samples and allow for adaptability.

• Instead of providing exact numbers for qualitative samples, authors can consider providing a range to better account for the variability across contexts.

• Developing a broad coding framework will be helpful as it will avoid redoing the process of in-depth analysis altogether and reduce redundancy.

• As the author mentions, that- “the goal is not national prevalence estimation but generation of transferable lessons on service delivery processes and system constraints”, authors should consider analysing variations within and across sites.

o There will be site-specific issues- for example, a barrier might be identified in majority of sites. However, the effect of this barrier on implementation might differ across sites.

o There will be variations in provider-centered and patient-centered perspectives. How do authors plan to deal with those variations before reaching conclusions, like relying on additional data or observations can be mentioned.

• The manuscript currently mentions concurrent analysis of quantitative and qualitative data. Authors should consider adding concurrent data collection and analysis in the manuscript where the authors mention conducting bracketing exercises. Evidence from implementation research underscores the value of this approach, as it facilitates the revising strategies and interview questions to better capture pertinent emerging data.

Minor Corrections

• Throughout the methodology, the study population is defined as women aged 18–49 years; however, the qualitative sampling section lists the age as 15–49 years. Please clarify the reason for this discrepancy.

• The final sentence of the Discussion section appears to be incomplete and requires correction.

**********

what does this mean?). If published, this will include your full peer review and any attached files.

If you choose “no”, your identity will remain anonymous but your review may still be made public.

**Do you want your identity to be public for this peer review?**  For information about this choice, including consent withdrawal, please see our Privacy Policy

Reviewer #1: No

Reviewer #2: No

**********

[NOTE: If reviewer comments were submitted as an attachment file, they will be attached to this email and accessible via the submission site. Please log into your account, locate the manuscript record, and check for the action link "View Attachments". If this link does not appear, there are no attachment files.]

To ensure your figures meet our technical requirements, please review our figure guidelines: https://journals.plos.org/plosone/s/figures

You may also use PLOS’s free figure tool, NAAS, to help you prepare publication quality figures: https://journals.plos.org/plosone/s/figures#loc-tools-for-figure-preparation.

NAAS will assess whether your figures meet our technical requirements by comparing each figure against our figure specifications.

# Author comment: Author response to Decision Letter 1

**DOI:** 10.1371/journal.pone.0358274.r003

23 Jul 2026

RESPONSE TO REVIEWERS

Editor Comment 1

Comment: Title: It is a multi center study and not multi centric study.

Response: Thank you for the observation. We have modified the Title as suggested.

Location of Revision: Page 1, Lines 1–2.

________________________________________

Editor Comment 2A

Comment: Why unmarried women are excluded. The risk of unsafe abortion and barriers to CAC among unmarried women are equally important.

Editor Comment 2B

Comment: Legally defined age is 18 to 49 but pregnancy below 18 years is not illegal per se. By excluding the 15-18 age group, in Indian context, a key and vulnerable beneficiary is getting excluded from the study.

Response: A & B. We thank the reviewer for this important observation and fully agree that unmarried women and women aged 15–18 years may experience substantial barriers to accessing safe abortion services and may be at increased risk of unsafe abortion. Their experiences and needs are highly relevant within the broader context of Comprehensive Abortion Care (CAC). However, the present study is designed primarily to examine CAC service access and utilization among women in rural areas which include certain tribal communities. Given the sensitive nature of abortion-related research in conservative rural and tribal settings, inclusion of unmarried women and women aged 15–18 years raised important operational and ethical considerations related to privacy, social stigma, community acceptability, and participant safety, particularly within household-based surveys. Inclusion of the unmarried women would require different recruitment strategies and confidentiality safeguards, which are beyond the scope of the present household survey design. Nevertheless, we acknowledge that exclusion of unmarried women and aged below 15-18 yrs may limit the comprehensiveness and generalizability of the findings. We have now explicitly acknowledged this as a limitation in the manuscript and highlighted the need for future research specifically focusing on the experiences, barriers, and care-seeking pathways of unmarried women and married women between 15-18 yrs seeking abortion services in rural India.

Location of Revision: Page 21, Lines 481–484, 488–492.

________________________________________

Editor Comment 2C

Comment: Sample size: Assuming 50% prevalence across all selected MRHRUs is an untenable value for sample size calculation. In absence of relevant literature, pilot study is undertaken to calculate sample size. If the results show very low prevalence say < 10% or very high prevalence, say >90%, the sample size estimation will become null and void.

Response: We thank the reviewer for these important observations. For the community-based survey, the sample size was calculated assuming a 50% prevalence of knowledge regarding safe abortion services, as no prior multicentric estimates from comparable rural Indian settings were available. A prevalence of 50% was chosen as a conservative assumption because it provides the maximum variability and therefore yields the largest sample size when prior prevalence estimates are unavailable. ( Daniel WW., 2013, Lwanga SK., 1991, Maple Tech IL., 2019, Pourhoseingholi, M. A.,2013). We agree that a pilot study can help generate preliminary estimates; however, pilot studies often involve relatively small samples and may not provide stable prevalence estimates for a large multi-center survey. Additionally, considering abortion is a sensitive subject, it’s not feasible to conduct a pilot study per say, we only have conducted the pilot testing of the tools to assess the clarity, comprehensibility, and contextual relevance. Considering the exploratory and implementation-focused nature of the study, we considered the use of a 50% prevalence assumption to be methodologically appropriate in the absence of relevant Indian literature.

Editor Comment 2D

Comment: What is the rationale for design effect of 2?

Response: A design effect (DE) 2 was applied because the study employs a multistage cluster sampling design across villages and study sites. Women within the same cluster may share similar socio-demographic characteristics and health-seeking behaviors resulting variance inflation compared to simple random sampling. In absence of prior ICC for knowledge of safe abortion we have used DE=2 as conservative design effect.

Editor Comment 2F

Comment: In a cross sectional survey, there is no utility of accounting for attrition.

Response: We thank the reviewer for this important observation and agree that attrition is generally not applicable in a cross-sectional survey, as there is no follow-up of participants over time. In the present study, the additional 10% was included to account for potential non-response rather than attrition. Given the sensitivity of this subject , some eligible participants may decline participation or may not feel comfortable responding during household-based interviews. To address this possibility and ensure adequate sample size, a conservative non-response rate of 10% was considered. The manuscript has now been revised to clarify this distinction.

Location of Revision: Page no – 16, Line no- 361-363

Editor Comment 2G

Comment:For qualitative component, age range is described as 15 to 49, contradicting the legal age of 18 to 49 years

Response:Thank you for pointing this out. This was a typographical error in the qualitative component description. The age range has been corrected to 18–49 years to maintain consistency throughout the protocol

Location of Revision: Page no – 16; Line no- 364-366

Editor Comment 2H

Comment:How can multivariable regression models be generated if there are no a priori hypotheses stated and sample sizes are calculated as per hypotheses?

Response:We thank the reviewer for this observation. We agree that, given the primarily descriptive and exploratory nature of the study, no a priori hypotheses were specified for these associations.

In light of the reviewer’s comment, we have removed the prespecified multivariable regression analysis from the protocol. If the final dataset provides sufficient observations and variability in the outcomes of interest, we may consider conducting additional exploratory analyses during the data analysis phase. Any such analyses will be clearly identified as exploratory and interpreted with appropriate caution.

The relevant section of the manuscript has been revised accordingly.

Location of Revision: Page no – 16; Line no- 355-358

Editor Comment 2I

Comment:How missing data will be handled, should be explained.

Response:Missing data will be assessed for each variable. For the primary outcome, complete case analysis will be used if missingness data is below 5%; otherwise, multiple imputation by chained equations (MICE) will be performed under the missing at random assumption

Location of Revision: Page no – 17; Line no- 360-362

________________________________________

Reviewer 1

Comment 1: Elaborate more on the selection and deployment of data collectors/ interviewers/ moderators for qualitative data collection - what kind of relationship do they share with the community/ what training they have undergone/ what stakeholders they will be engaging with to make them suitable to undertake such sensitive conversations in their respective areas? This may also include their relevant past experience in the same area.

Response: We thank the reviewer for this useful suggestion. We agree that additional information regarding the selection, training, and deployment of qualitative data collectors is important, particularly given the sensitive nature of the study topic. We have revised the methodology section to provide additional details on the selection, training, and deployment of qualitative data collection teams. 1. A multi-level capacity-building approach will be adopted for the selection and deployment of qualitative data collection teams across study sites. In the first phase, Site Principal Investigators (PIs) from all participating sites will undergo sensitization through a two-day in-person Training of Trainers (ToT) workshop. Subsequently, Site PIs will recruit Project Technical Staff (PTS) at their respective sites, for whom prior experience in conducting qualitative research will be a prerequisite for recruitment.

Training will be delivered by a multidisciplinary team with substantial expertise in sensitive research areas, including reproductive health and maternal mental health, with backgrounds in community medicine, social anthropology, and ethnographic research.

Location of Revision: Page 14, Lines 279–300.

Comment 2: How are the live-in relationships in Maharashtra area being defined? Is there a time-based criterion for inclusion, or any criteria? The exception has only been mentioned, not explained.

Response: We have revised the manuscript to clarify the definition and eligibility criteria for inclusion of women in such unions at the Maharashtra study site. We thank the reviewer for this important observation. For the Maharashtra study site, eligibility will also extend to women who are co-habiting with their partners in live-in which is a socially acceptable local customary practice in this area. These women self-identify as married, acknowledging the contextual specificity of marital norms in this setting. For example, among the Warli community represented at the Dahanu study site, customary union arrangements such as ‘Gharori’ or ‘Khandadhya’ are socially recognized and functionally equivalent to marriage.(Gujrat Tribal Research Training Society)

No specific time-based criterion has been applied. Eligibility is based on self-identification as married within a socially recognized union, as the objective is to capture women who are socially and functionally part of a marital relationship within the local context, irrespective of the duration of cohabitation.

Location of Revision: Page 9, Lines 172-177.

Comment 3: How were the questions for moderator/ facilitator guide(s) for KII and FGDs arrived at? Was the CFIR framework used for the design of the guide(s) as well? What were the key literature findings that guided the choice of questions.

Response: The IDI and FGD guides were developed using a systematic and rigorous approach informed by an extensive literature review, consultations with national-level experts in the subject area, and qualitative research. Key literature findings that informed guide development included decision-making processes, information pathways, care-seeking trajectories, quality of care, stigma, and other contextual barriers and facilitators influencing access and experiences of care. The CFIR framework also informed the development of the guides to ensure that relevant implementation domains and contextual factors were adequately captured.

Location of Revision: Page no 13; Line no 269-278

Comment 4: It is a good practice in qual research for moderator/ facilitators to reconvene after FGDs to tweak the guide(s) based on their subsequent experiences of FGDs. Some new areas may come up during FGD, which make good addition to the guide for subsequent discussions. While the authors mention maintaining an audit trail throughout the study, it is not clear of the same extends to this specific aspect of refining the tool.

Response: Thank you for highlighting this. Regular debriefing and reflective discussions were will be conducted throughout data collection to review field experiences and refine discussion guides where necessary, with modifications documented as part of the audit trail. Following this suggestion, we have added a sentence in the manuscript to clarify this process.

The exact sentence added is –

“Similarly, regular debriefing and reflective discussions will be conducted throughout data collection to refine discussion guides where necessary. All modifications in the study tool will also be documented in the audit trail.”

Location of Revision: Page no 15; Line no- 320-323

________________________________________

Reviewer 2

Comment 1: Predicting in advance, how much data will be enough is a requirement for a-priori sample size calculation and funding requirements. While it is mentioned that sampling will be guided by thematic saturation, the authors must be aware and mention somewhere in the section on “Qualitative sampling” that recruitment methods will facilitate varied samples and allow for adaptability.

Response: Thank you for this important suggestion. We have now added a sentence in the manuscript that the

“Final sample size will be guided by thematic saturation and contextual requirements across sites. This recruitment method will facilitate varied samples and allow for adaptability.”

Location of Revision: Page 11, Lines 229, 232–233.

Comment 2: Instead of providing exact numbers for qualitative samples, authors can consider providing a range to better account for the variability across contexts.

Response: We thank the reviewer for this suggestion. We have revised the qualitative sampling table to present indicative ranges rather than fixed sample sizes for participant groups where recruitment may vary across contexts. We have also added a note clarifying that final sample sizes will be guided by participant availability and thematic saturation.

Location of Revision: Page no 11-12 ; Table 2

Comment 3: Developing a broad coding framework will be helpful as it will avoid redoing the process of in-depth analysis altogether and reduce redundancy.

Response: Thank you for this valuable suggestion. We agree that developing a broad coding framework is important to ensure consistency across sites, reduce redundancy, and streamline the analytic process. We have now clarified in the manuscript the same-

“An initial broad coding framework will be developed to guide analysis across sites and participant groups and will be iteratively refined throughout the analytic process to incorporate emerging themes and contextual variations.”

Location of Revision: Page no 17, Line 378-381

Comment 4: As the author mentions, that- “the goal is not national prevalence estimation but generation of transferable lessons on service delivery processes and system constraints”, authors should consider analysing variations within and across sites.

Response: We thank the reviewer for this valuable suggestion and agree that examining variations within and across sites is important. As noted in the manuscript, data will be analysed at both the overall and site-specific levels. This will enable assessment of site-level variations in facility readiness, provider knowledge, women's awareness, service utilization, and experiences related to Comprehensive Abortion Care services.

Location of Revision: Page no: 15; Line no: 329-333

Comment 5: There will be site-specific issues- for example, a barrier might be identified in majority of sites. However, the effect of this barrier on implementation might differ across sites.

Response: We thank the reviewer for this valuable suggestion. We agree that, barriers might be common across the sites, however the effect of this barrier on implementation might differ across sites. In the manuscript, we mentioned that the analysis would be at overall and site-specific levels. Now in the manuscript we have further elaborated that in addition to identifying common barriers and facilitators, we also explore how these factors vary across sites.

Location of Revision: Page no: 15; Line no: 329-333

Comment 6: There will be site-specific issues- for example, a barrier might be identified in majority of sites. However, the effect of this barrier on implementation might differ across sites.

Response: We thank the reviewer for this valuable suggestion. We agree that, barriers might be common across the sites, however the effect of this barrier on implementation might differ across sites. In the manuscript, we mentioned that the analysis would be at overall and site-specific levels. Now in the manuscript we have further elaborated that in addition to identifying common barriers and facilitators, we also explore how these factors vary across sites.

Location of Revision: Page no: 15; Line no: 329-333

Comment 7: There will be variations in provider-centered and patient-centered perspectives. How do authors pl

Attachment Submitted filename: Editorial comments.docx

# Peer review: Decision Letter 1

**DOI:** 10.1371/journal.pone.0358274.r004

**Authors:** Arun Kumar Sharma

19 Aug 2026

Dear Dr. Mukerjee,

Please submit your revised manuscript by Oct 03 2026 11:59PM. If you will need more time than this to complete your revisions, please reply to this message or contact the journal office at plosone@plos.org. When you're ready to submit your revision, log on to https://www.editorialmanager.com/pone/ and select the 'Submissions Needing Revision' folder to locate your manuscript file.

- A letter that responds to each point raised by the academic editor and reviewer(s). You should upload this letter as a separate file labeled 'Response to Reviewers'.
- A marked-up copy of your manuscript that highlights changes made to the original version. You should upload this as a separate file labeled 'Revised Manuscript with Track Changes'.
- An unmarked version of your revised paper without tracked changes. You should upload this as a separate file labeled 'Manuscript'.

If you would like to make changes to your financial disclosure, please include your updated statement in your cover letter. Guidelines for resubmitting your figure files are available below the reviewer comments at the end of this letter.

If applicable, we recommend that you deposit your laboratory protocols in protocols.io to enhance the reproducibility of your results. Protocols.io assigns your protocol its own identifier (DOI) so that it can be cited independently in the future. For instructions see: https://journals.plos.org/plosone/s/submission-guidelines#loc-laboratory-protocols. Additionally, PLOS One offers an option for publishing peer-reviewed Lab Protocol articles, which describe protocols hosted on protocols.io. Read more information on sharing protocols at https://plos.org/protocols?utm_medium=editorial-email&utm_source=authorletters&utm_campaign=protocols.

As the corresponding author, your ORCID iD is verified in the submission system and will appear in the published article. PLOS supports the use of ORCID, and we encourage all coauthors to register for an ORCID iD and use it as well. Please encourage your coauthors to verify their ORCID iD within the submission system before final acceptance, as unverified ORCID iDs will not appear in the published article. *Only*  the individual author can complete the verification step; PLOS staff *cannot*  verify ORCID iDs on behalf of authors.

We look forward to receiving your revised manuscript.

Kind regards,

Arun Kumar Sharma

Academic Editor

PLOS One

Journal Requirements:

If the reviewer comments include a recommendation to cite specific previously published works, please review and evaluate these publications to determine whether they are relevant and should be cited. There is no requirement to cite these works unless the editor has indicated otherwise.

Additional Editor Comments :

All concerns of the reviewers have been addressed satisfactorily sans one comment of the editor.

The design effect is calculated using the formula, assuming equal cluster sizes: 1+(m-1)ρ where m= average number of individuals sampled per cluster. ρ= intracluster correlation coefficient. In case of unequal cluster sizes, following formula is used: design effect=1+[m(1+CV*2)-1]ρ

An arbitrary design effect is statistically not tenable.

I understand that the project has been approved by ICMR and modifications are not possible. Nonetheless, for the sake of the readers of the journal, it is pertinent to mention how the effect size was determined.

Authors may consider explaining that design effect was arbitrary.

[Note: HTML markup is below. Please do not edit.]

Reviewers' comments:

Reviewer's Responses to Questions

**Comments to the Author**

1. Does the manuscript provide a valid rationale for the proposed study, with clearly identified and justified research questions?

Reviewer #1: Yes

Reviewer #2: Yes

**********

2. Is the protocol technically sound and planned in a manner that will lead to a meaningful outcome and allow testing the stated hypotheses?

Reviewer #1: Yes

Reviewer #2: Yes

**********

3. Is the methodology feasible and described in sufficient detail to allow the work to be replicable?

Reviewer #1: Yes

Reviewer #2: Yes

**********

4. Have the authors described where all data underlying the findings will be made available when the study is complete?

The PLOS Data policy

Reviewer #1: Yes

Reviewer #2: Yes

**********

5. Is the manuscript presented in an intelligible fashion and written in standard English?

Reviewer #1: Yes

Reviewer #2: Yes

**********

Please use the space provided to explain your answers to the questions above and, if applicable, provide comments about issues authors must address before this protocol can be accepted for publication. You may also include additional comments for the author, including concerns about research or publication ethics.

You may also provide optional suggestions and comments to authors that they might find helpful in planning their study.

Reviewer #1: All the reviewer comments have been addressed satisfactorily. The authors have provided a detailed explanation, and comprehensive logs of changes made.

Manuscript recommended to proceed for next steps.

Reviewer #2: Including the suggestions from the editor and reviewers, the manuscript can now be accepted for publication.

**********

what does this mean?). If published, this will include your full peer review and any attached files.

If you choose “no”, your identity will remain anonymous but your review may still be made public.

**Do you want your identity to be public for this peer review?**  For information about this choice, including consent withdrawal, please see our Privacy Policy

Reviewer #1: No

Reviewer #2: No

**********

[NOTE: If reviewer comments were submitted as an attachment file, they will be attached to this email and accessible via the submission site. Please log into your account, locate the manuscript record, and check for the action link "View Attachments". If this link does not appear, there are no attachment files.]

To ensure your figures meet our technical requirements, please review our figure guidelines: https://journals.plos.org/plosone/s/figures

You may also use PLOS’s free figure tool, NAAS, to help you prepare publication quality figures: https://journals.plos.org/plosone/s/figures#loc-tools-for-figure-preparation.

NAAS will assess whether your figures meet our technical requirements by comparing each figure against our figure specifications.

# Author comment: Author response to Decision Letter 2

**DOI:** 10.1371/journal.pone.0358274.r005

27 Aug 2026

Response to Editor

Additional Editor Comments:

All concerns of the reviewers have been addressed satisfactorily sans one comment of the editor.

The design effect is calculated using the formula, assuming equal cluster sizes: 1+(m-1)ρ where m= average number of individuals sampled per cluster. ρ= intracluster correlation coefficient. In case of unequal cluster sizes, following formula is used: design effect=1+[m(1+CV*2)-1]ρ

An arbitrary design effect is statistically not tenable.

I understand that the project has been approved by ICMR and modifications are not possible. Nonetheless, for the sake of the readers of the journal, it is pertinent to mention how the effect size was determined.

Authors may consider explaining that the design effect was arbitrary.

Response

We thank the Editor for this important methodological observation. We agree that the design effect of 2 used in the protocol was not derived empirically from an estimated intracluster correlation coefficient (ICC) and average cluster size. Rather, it was specified a priori as a conservative planning assumption for the sample-size calculation, given the cluster-based study design and the absence of reliable prior ICC estimates for the study setting and outcome at the time of protocol development.

We have now clarified this in the manuscript to ensure transparency for readers. We have also explicitly stated that the design effect of 2 was a conservative a priori assumption used for sample-size planning, rather than an empirically estimated parameter.

Line and Page no: Page no 10; Line no: 198-199

2. Thank you for your message. We have carefully reviewed the manuscript and confirm that there is no funding-related information included in any section of the manuscript.

Attachment Submitted filename: RESPONSE TO REVIEWERS_V4.docx

# Editor report: Decision Letter 2

**DOI:** 10.1371/journal.pone.0358274.r006

**Authors:** Arun Kumar Sharma

30 Aug 2026

Status, Barriers and Facilitators of Comprehensive Abortion Care Services in rural  India: A Multicenter Mixed-Methods Study Protocol

PONE-D-26-10674R2

Dear Dr. Mukherjee,

We’re pleased to inform you that your manuscript has been judged scientifically suitable for publication and will be formally accepted for publication once it meets all outstanding technical requirements.

Within one week, you’ll receive an e-mail detailing the required amendments. When these have been addressed, you’ll receive a formal acceptance letter and your manuscript will be scheduled for publication.

An invoice will be generated when your article is formally accepted. Please note, if your institution has a publishing partnership with PLOS and your article meets the relevant criteria, all or part of your publication costs will be covered. Please make sure your user information is up-to-date by logging into Editorial Manager at Editorial Manager® and clicking the ‘Update My Information' link at the top of the page. For questions related to billing, please contact billing support.

If your institution or institutions have a press office, please notify them about your upcoming paper to help maximize its impact. If they’ll be preparing press materials, please inform our press team as soon as possible -- no later than 48 hours after receiving the formal acceptance. Your manuscript will remain under strict press embargo until 2 pm Eastern Time on the date of publication. For more information, please contact onepress@plos.org.

Kind regards,

Arun Kumar Sharma

Academic Editor

PLOS One

Additional Editor Comments (optional):

The response to the comments is satisfactory.

Reviewers' comments:

# Editor report: Acceptance letter

**DOI:** 10.1371/journal.pone.0358274.r007

**Authors:** Arun Kumar Sharma

PONE-D-26-10674R2

PLOS One

Dear Dr. Mukerjee,

I'm pleased to inform you that your manuscript has been deemed suitable for publication in PLOS One. Congratulations! Your manuscript is now being handed over to our production team.

At this stage, our production department will prepare your paper for publication. This includes ensuring the following:

* All references, tables, and figures are properly cited

* All relevant supporting information is included in the manuscript submission,

* There are no issues that prevent the paper from being properly typeset

You will receive further instructions from the production team, including instructions on how to review your proof when it is ready. Please keep in mind that we are working through a large volume of accepted articles, so please give us a few days to review your paper and let you know the next and final steps.

Lastly, if your institution or institutions have a press office, please let them know about your upcoming paper now to help maximize its impact. If they'll be preparing press materials, please inform our press team within the next 48 hours. Your manuscript will remain under strict press embargo until 2 pm Eastern Time on the date of publication. For more information, please contact onepress@plos.org.

You will receive an invoice from PLOS for your publication fee after your manuscript has reached the completed accept phase. If you receive an email requesting payment before acceptance or for any other service, this may be a phishing scheme. Learn how to identify phishing emails and protect your accounts at https://explore.plos.org/phishing.

If we can help with anything else, please email us at customercare@plos.org.

Thank you for submitting your work to PLOS One and supporting open access.

Kind regards,

PLOS One Editorial Office Staff

on behalf of

Professor Arun Kumar Sharma

Academic Editor

PLOS One
